# Development of loop-mediated isothermal amplification (LAMP) combined with a lateral flow dipstick DNA biosensor for detection of α thalassemia 1 [Southeast Asia (SEA), ^--SEA^ deletion]

**DOI:** 10.1371/journal.pone.0360555

**Published:** 2026-10-12

**Authors:** Jutturong Ckumdee, Sataron Karnjanaopas, Duantida Songdej, Noppawan Tungbuppha, Thongchai Kaewphinit, Thanwa Wongsuk, Chanita Sanyear

**Affiliations:** 1 Department of Clinical Pathology, Faculty of Medicine Vajira Hospital, Navamindradhiraj University, Bangkok, Thailand; 2 Department of Pathology, Faculty of Medicine, Prince of Songkla University, Songkhla, Thailand; 3 Department of Pediatrics, Faculty of Medicine Ramathibodi Hospital, Mahidol University, Bangkok, Thailand; 4 Innovative Learning Center, Srinakharinwirot University, Thailand; Shoklo Malaria Research Unit, THAILAND

## Abstract

This study aimed to develop a one-step loop-mediated isothermal amplification combined with a lateral flow dipstick (LAMP-LFD) DNA biosensor for the detection of α^0^-thalassemia (SEA deletion). Genomic DNA was obtained from 48 α^0^-thalassemia (SEA deletion)-positive and 48 (SEA deletion)-negative (normal) Thai subjects. DNA was analyzed by LAMP assay at 61°C for 60 min using specific primers for the α^0^-thalassemia (SEA deletion) type. Following amplification, the products were visually detected using an LFD, with results available within 5–10 min. The one-step LAMP-LFD DNA biosensor could detect DNA carrying the α^0^-thalassemia (SEA deletion) type at levels as low as 200 pg, with 100% sensitivity and 100% specificity. Furthermore, no cross-reactivity was observed with DNA carrying other α^0^-thalassemia deletion types (THAI deletion). The LAMP-LFD DNA biosensor is useful for identifying carriers of α^0^-thalassemia (SEA deletion) (the most common deletion type found in Thailand and across Southeast Asia). This novel one-step LAMP-LFD kit will be beneficial for medical laboratory technologists performing genetic carrier screening tests as it is simple to perform and requires minimal equipment and time. Furthermore, this method supports clinicians in providing genetic counseling and implementing preventive strategies against severe thalassemia, a significant public health concern in Thailand.

## Introduction

α-Thalassemia-1 Southeast Asia (SEA) deletion type (α^0^-thalassemia) is the most common inherited blood disorder found in the Southeast Asian population, characterized by the deletion of both α-globin genes on a single chromosome [1–5]. Couples wherein both partners carry the α^0^-thalassemia genotype, particularly the SEA deletion, are at risk of having a baby affected with Hb Bart’s hydrops fetalis syndrome (a severe form of α-thalassemia caused by the complete deletion of all four α-globin genes, resulting in fetal or neonatal death) [2,6,7]. Thus, DNA analysis is required for identifying α^0^-thalassemia, which is an important step in genetic counseling and in the prevention and control of thalassemia among at-risk families [8–10]. Several molecular techniques are used for detecting α^0^-thalassemia, including conventional polymerase chain reaction (PCR), multiplex PCR, and real-time PCR [5,8,9,11–13]. Recently, a rapid molecular technique based on loop-mediated isothermal amplification (LAMP) colorimetric assay has been used to determine α^0^-thalassemia, including SEA and THAI deletions [14]. LAMP amplifies DNA at a constant temperature of 60°C–65°C [15]. The amplified products are typically detected by agarose gel electrophoresis, followed by ethidium bromide staining and ultraviolet transillumination. However, LAMP uses multiple primers, which generates a complex mixture of DNA fragments of varying sizes. Thus, gel analysis alone is unreliable for distinguishing specific from nonspecific amplification products. For this reason, additional confirmation by restriction endonuclease digestion [15] or hybridization to specific probes is recommended to reduce the risk of false positive results [16]. Previous studies have used LAMP assay for rapidly detecting various pathogens, and the results are visualized through colorimetric, lateral flow, turbidity, and Gap-LAMP methods [17–21,25]. In the present study, a one-step LAMP combined with a lateral flow dipstick (LFD) DNA biosensor was developed to determine the α^0^-thalassemia (SEA deletion) type, with results being reported within 1 h.

## Materials and methods

### Ethics statement

Ethical approval for the study was granted by the Ethics Committee of the Institutional Review Board at the Faculty of Medicine Vajira Hospital, Navamindradhiraj University, Bangkok, Thailand (Certificate of Exemption: COE 026/2023 X; Date of Acknowledgement: 05/09/2023).

### Subjects and DNA analysis

Following ethical approval, leftover DNA specimens from 48 α^0^-thalassemia (SEA deletion)-positive and 48 (SEA deletion)-negative (normal) Thai subjects were collected from the Department of Pathology, Faculty of Medicine, Prince of Songkla University, Thailand, during the period of June to December 2024. All specimens had been previously characterized by PCR, and genomic DNA was extracted from peripheral blood leukocytes using a standard commercial kit (Geneaid™ DNA Isolation Kit).

### LAMP Primers

LAMP primers for α^0^-thalassemia (SEA deletion) type were designed based on the published genomic sequence of the α-thalassemia breakpoint region specific for the α^0^-thalassemia (SEA deletion) genome (Homo sapiens alpha thalassemia breakpoint region genomic sequence, GenBank: KX458114.1) using PrimerExplorer version 5 (http://primerexplorer.jp/elamp4.0.0/index.html). All primers, including the biotin-labeled BIP primer and LB-FITC, were synthesized by Bio Basic Inc. (Canada), and all details are provided in Fig 1 and Table 1 [14].

**Fig 1:**
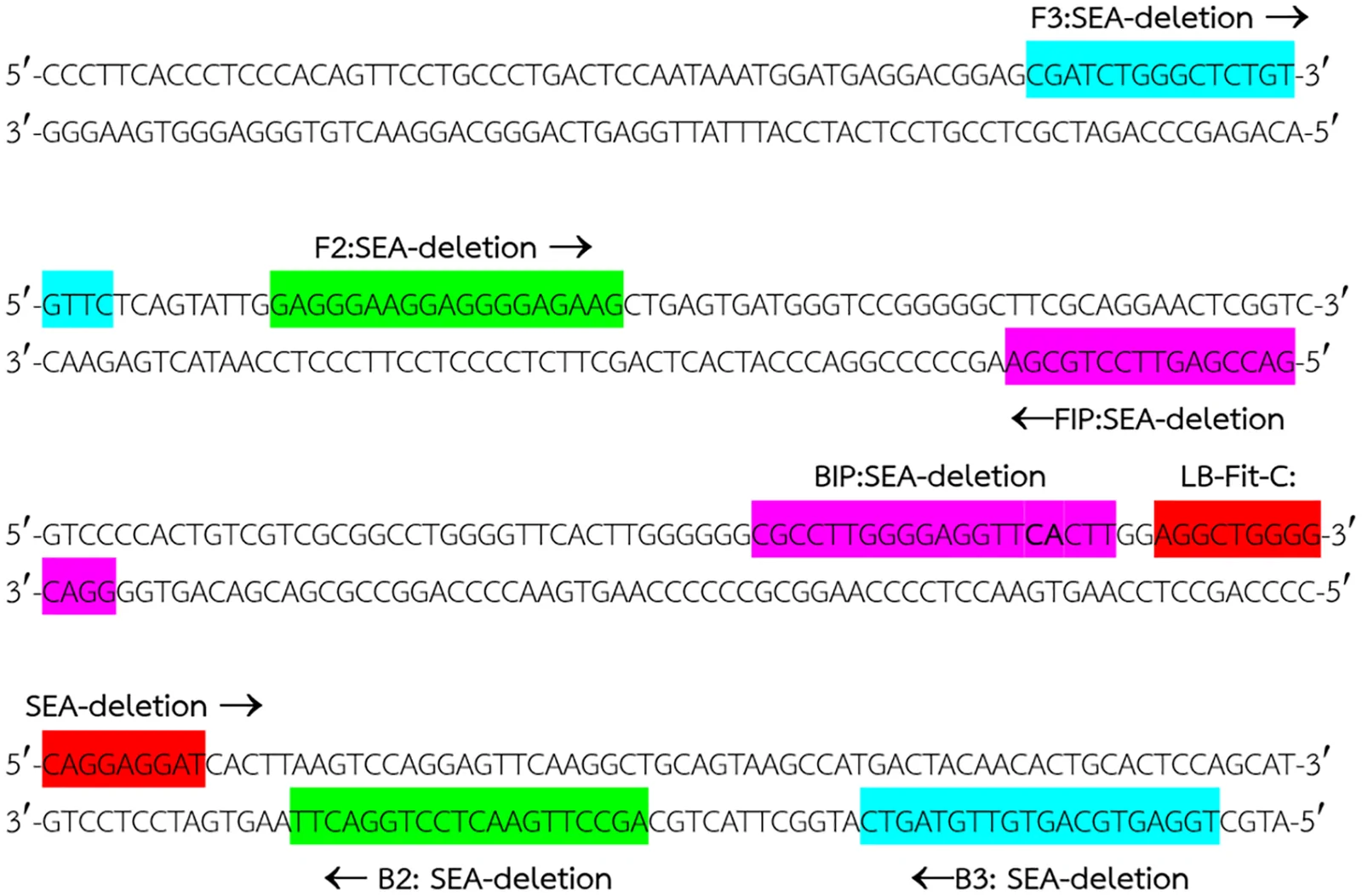
Nucleotide sequence of the α thalassemia breakpoint region gene specific for α^0^ -thalassemia, SEA deletion genome (GenBank: KX458114.1). The positions and orientations of the LAMP primer set comprising F3 (light blue), B3 (light blue), FIP (F1c (pink) -TTT-F2 (green), biotinylated BIP (B1c (pink) -TTT-B2 (green), and the FITC-labeled LB probe (red) are indicated by arrows and highlighted sequences.

**Table 1:** Primers and LB-FITC probe used for LAMP of the α-thalassemia breakpoint region gene specific for α^0^-thalassemia, SEA deletion (GenBank: KX458114.1). The primer and probe sequences are listed [14].

Primers name	Sequences (5′ – 3′)
F3	CGATCTGGGCTCTGTGTTC
B3	TGGAGTGCAGTGTTGTAGTC
FIP (F1c/TTT/F2)	GGACGACCGAGTTCCTGCGA- TTT-GAGGGAAGGAGGGGAGAAG
BIP (B1c/TTT/B2)	Biotin/Dig-CGCCTTGGGGAGGTTCACTT- TTT-AGCCTTGAACTCCTGGACTT
LB-Fit-C (probe)	FITC- AGGCTGGGGCAGGAGGAT

### LAMP amplification and dipstick detection

LAMP reactions, which are widely used for microbial detection, were performed in accordance with the method of Notomi et al. [15]. Amplification was conducted in the range of 59°C–65°C for 60 min. LAMP products were detected using the LFD DNA biosensor, allowing visual interpretation within 10–15 min. All LAMP reactions were performed in a total volume of 25 μL, containing 2 μM each of FIP and BIP primers, 0.2 μM each of F3 and B3 primers, and 0.2 μM of 5′-fluorescein isothiocyanate (FITC)-labeled DNA probe. The optimal concentration of dNTP Mix was 1.2 mM (Promega, Madison, WI, USA), and the optimal MgSO_4_ concentration was 6 mM. The reaction mixture also contained 0.5 M betaine (Sigma-Aldrich, St. Louis, MO, USA), 1 U of Bst DNA polymerase (large fragment; New England Biolabs Inc., Beverly, MA, USA), and 1 × supplied buffer. After the addition of genomic DNA from α^0^-thalassemia (SEA deletion) and overlaid with 20 µl mineral oil. The reaction mixture was incubated at 61°C for 1 h. Subsequently, 5 μL of the LAMP product was mixed with 100 μL of assay buffer. Then, an LFD was immersed in the mixture for approximately 10–15 min.

### LFD assay

To prepare gold nanoparticle-labeled antibodies, anti-FITC antibodies were passively adsorbed onto 20 nm colloidal gold under neutral pH conditions. Following bovine serum albumin blocking for conjugate stabilization, the particles were harvested via centrifugation and resuspended in gold dilution buffer prior to use. Based on a previously reported fabrication protocol, the lateral flow test strip was constructed by mounting a sample pad, a conjugate pad impregnated with gold nanoparticle-tagged anti-FITC antibodies, a nitrocellulose membrane, and an absorbent pad onto a backing support [28]. For the lateral flow strip construction, test and control lines were formed on a CN140 nitrocellulose membrane (Sartorius, Germany) by dispensing anti-biotin antibody (1.0 mg/mL in PBS) and goat anti-mouse IgG (1.0 mg/mL in PBS), respectively, at a flow rate of 1 μL/cm. Following incubation at 37 °C for drying, the membrane was ready for assembly. Separately, anti-FITC antibody-gold nanoparticle conjugates were applied to the GF33 glass fiber conjugate pad (Cytiva, USA) at an optical density of 15 and thoroughly dried prior to strip assembly. To facilitate uninterrupted capillary flow, all strip components were aligned with an overlap of approximately 2 mm between adjacent materials. The assembled strips were then dried at 37 °C and preserved in desiccant-containing sealed aluminum foil pouches prior to use. Sensor fabrication was executed by K.BIO SCIENCES Co., Ltd. (Pathum Thani, Thailand) under an ISO 13485-certified quality system, following the authors’ specifications and an established lateral flow biosensor manufacturing protocol [28].

An FITC-labeled oligonucleotide probe was synthesized by Bio Basic Inc. (Canada). This probe was specifically designed to hybridize with a segment of the specific α-thalassemia breakpoint region gene previously identified as being associated with α^0^-thalassemia (SEA deletion). The target region is situated between the binding sites of B1c and B2 primers. In accordance with protocols established in previous diagnostic assays, 0.2 μM of the FITC-labeled probe (designated LB-FITC, 5′-AGGCTGGGGCAGGAGGAT-3′) was introduced at the initial stage of the reaction, thereby obviating the requirement for post-LAMP hybridization steps. Following amplification, the LAMP product was combined with 100 μL of assay buffer. Subsequently, a customized LFD, developed by K.BIO SCIENCES CO., LTD. (Thailand), was inserted into the reaction mixture and incubated at ambient temperature for 5–10 min. The presence of hybridization signals was then visually detected by the unaided eye [22].

### Sensitivity and specificity of one-step LAMP-LFD detection

To determine the sensitivity, 10-fold serial dilutions (10^−1^ to 10^−6^) of genomic DNA from α^0^-thalassemia (SEA deletion) samples, prepared from 20 ng of DNA, were used as templates for the one-step LAMP-LFD assay under optimized conditions. To determine the specificity, 20 ng of genomic DNA extracted from α^0^-thalassemia (SEA deletion) samples and other α-thalassemia deletion types, including the THAI deletion, were used as templates for the one-step LAMP-LFD assay under the same conditions. The biotin- and FITC-labeled LAMP products were subsequently analyzed using LFD.

### Testing of clinical samples

A total of 96 clinical samples [48 α^0^-thalassemia (SEA deletion)-positive and 48 α^0^-thalassemia (SEA deletion)-negative samples] were analyzed in triplicate using the one-step LAMP-LFD assay under optimized conditions and compared with the results obtained by standard PCR.

### Statistical analysis

We evaluated the performance of our LAMP-LFD assay against standard PCR using a 2 x 2 contingency table. We calculated sensitivity, specificity, positive predictive value (PPV), negative predictive value (NPV), and overall accuracy, with 95% confidence intervals (CI) determined via the Wilson/Brown method. Inter-method agreement was assessed using Cohen’s kappa coefficient (κ) interpreted per Landis and Koch criteria. We analyzed categorical associations using Fisher’s exact test, considering P < 0.05 statistically significant. All analyses and CI calculations were performed in GraphPad Prism version 10.0 (GraphPad Software, San Diego, CA, USA).

## Results

### Optimization of LAMP reaction conditions

When LAMP was performed at 59°C, 61°C, 63°C, and 65°C for 60 min using 20 ng of genomic DNA from α^0^-thalassemia (SEA deletion) samples, all four temperatures tested generated similar LAMP product patterns. However, the clearest and strongest bands were observed at 61°C. Therefore, 61°C was selected as the optimal temperature and used for all subsequent assays in this study. The MgSO_4_ concentration was optimized for LAMP assay to detect specific α^0^-thalassemia (SEA deletion) gene mutations. Amplification products were observed at MgSO_4_ concentrations of 2, 4, 6, 8, 10, and 12 mM. The LAMP reaction was performed using 4 mM MgSO_4_ under the specified conditions. The resulting LAMP products were analyzed using 2% agarose gel electrophoresis. LAMP reactions were performed using tubes containing dNTP concentrations ranging from 0.4 mM to 1.6 mM. The optimal dNTP concentration for detecting specific α^0^-thalassemia (SEA deletion) gene mutations was determined to be 1.2 mM. Amplification products were observed at dNTP concentrations of 0.4, 0.8, 1.2, and 1.6 mM. Subsequently, the LAMP reaction was conducted using a dNTP concentration of 1.2 mM under the same monitoring conditions as previously described. The resulting LAMP products were analyzed using 2% agarose gel electrophoresis (Fig 2A-2C).

**Fig 2:**
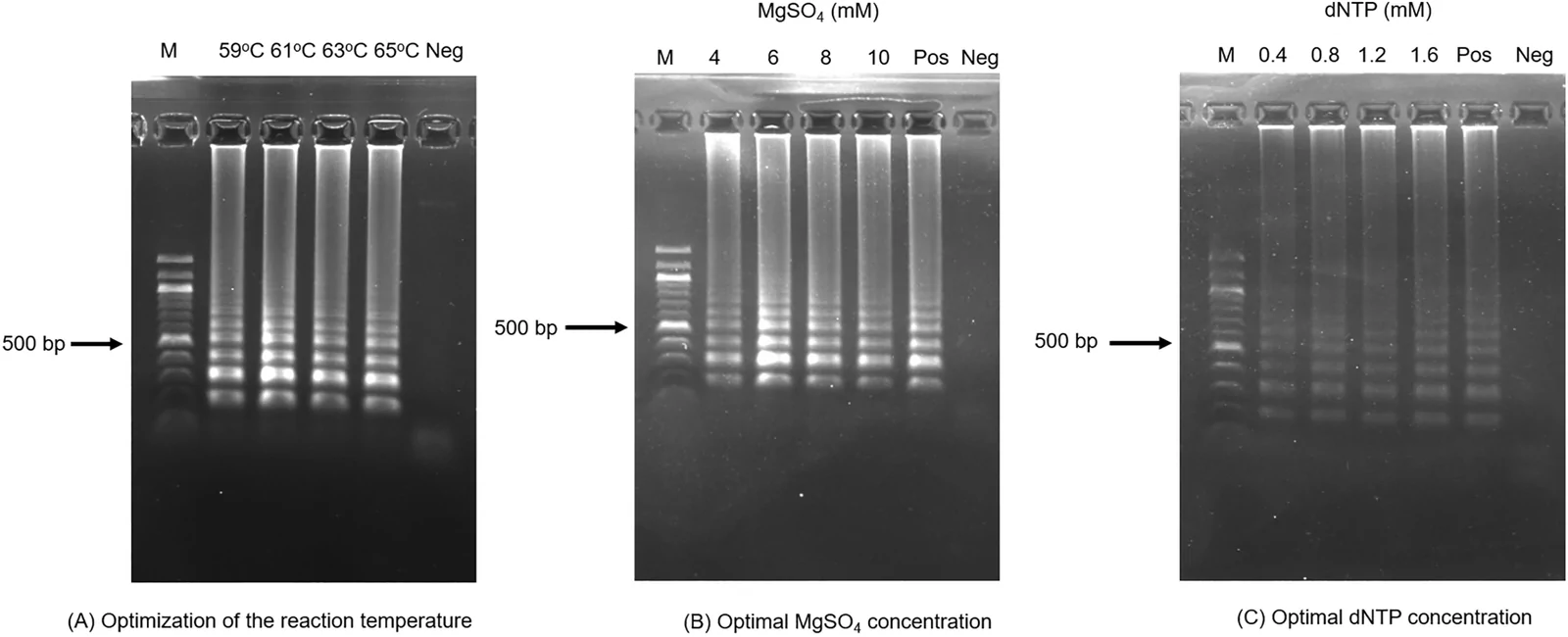
Optimization of LAMP reaction conditions. (A) Optimization of the reaction temperature for LAMP assay. Lane 1 (M) represents the DNA ladder marker; lanes 2–5 show LAMP reactions performed at 59°C, 61°C, 63°C, and 65°C, respectively; and lane 6 (Neg) represents the negative control. All LAMP reaction products were analyzed by 1.5% agarose gel electrophoresis. (B) Optimal MgSO_4_ concentration for LAMP assay. Lane 1 (M) represents the DNA ladder marker; lanes 2–5 show LAMP reactions performed at 2, 4, 6, 8, and 10 mM MgSO_4_ for 60 min; lane 6 (Pos) presents the positive control; and lane 7 (Neg) represents the negative control. All LAMP reaction products were analyzed by 1.5% agarose gel electrophoresis. (C) Optimal dNTP concentration for LAMP assay. Lane 1 (M) represents the DNA ladder marker; lanes 2–5 show LAMP reactions performed at 0.4, 0.8, 1.2, and 1.6 mM dNTP for 60 min; lane 6 (Pos) presents the positive control; and lane 7 (Neg) represents the negative control. All LAMP reaction products were analyzed by 1.5% agarose gel electrophoresis.

### Optimum FITC-labeled DNA probe concentration

The FITC-labeled DNA probe concentration was optimized to maximize signal intensity and minimize background noise. Probe concentrations ranging from 0.1 μM to 0.4 μM were evaluated. A concentration of 0.2 μM produced the strongest and most clearly distinguishable test line without nonspecific signal development and was therefore selected for further experiments (Fig 3).

**Fig 3:**
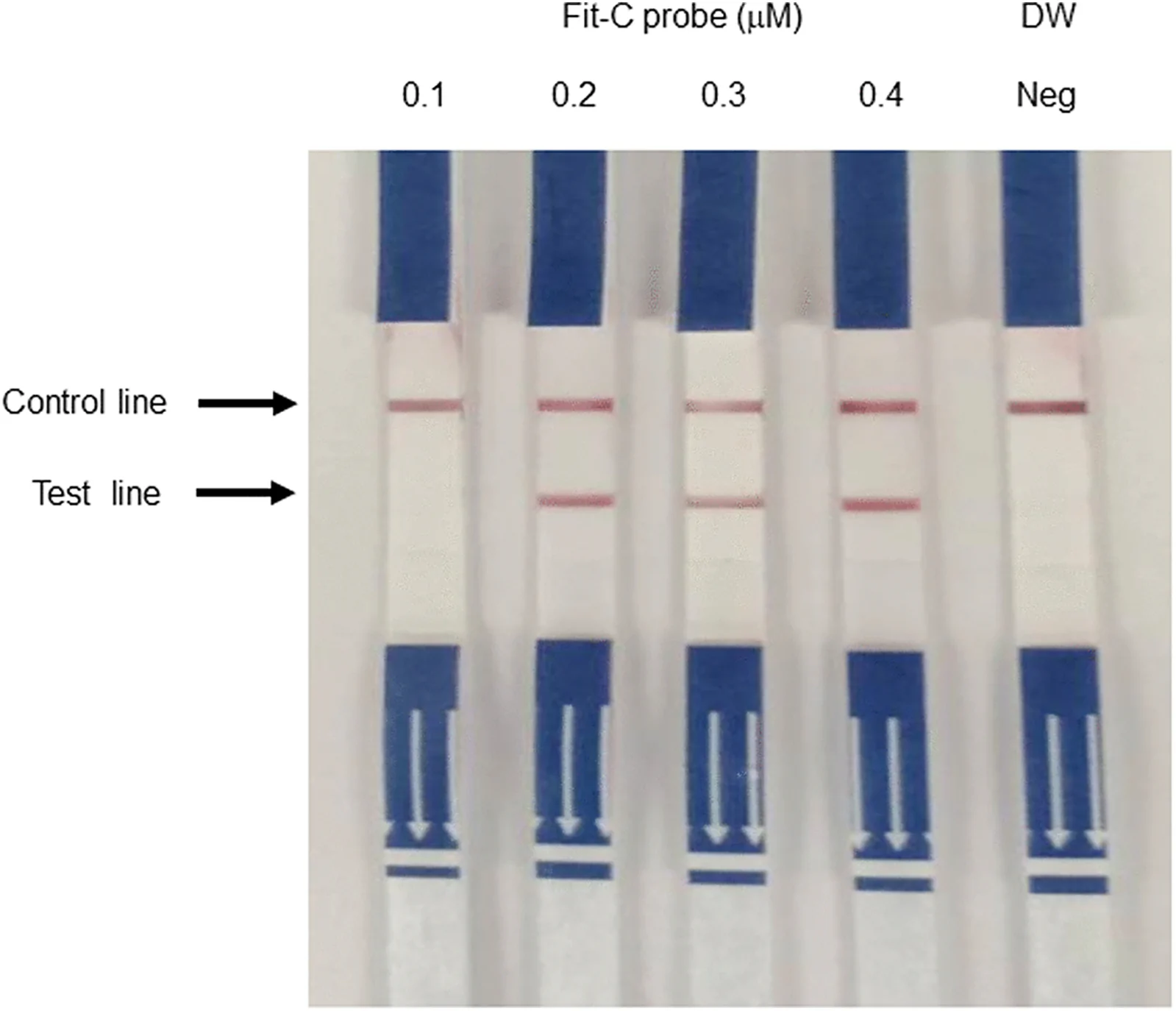
Optimum FITC-labeled DNA probe. Results of LAMP combined with a DNA probe and LFD DNA biosensor at different FITC-labeled DNA probe concentrations (0.1, 0.2, 0.3, and 0.4 μM) in the one-step LAMP-LFD reaction.

### Detection of one-step LAMP products using LFD DNA biosensor

LAMP amplicons were successfully detected using the LFD DNA biosensor without the need for gel electrophoresis. LFD strips were optimized by varying conjugated probe concentration, test/control line positioning on the nitrocellulose membrane, and buffer composition to minimize background. The final layout was chosen for clear test-line visibility and reproducible results across replicates. Positive samples produced two visible bands corresponding to the test and control lines, whereas negative controls showed only the control line. The LFD-based detection results were fully concordant with the results of agarose gel electrophoresis analysis (Fig 4), confirming the reliability of visual detection.

**Fig 4:**
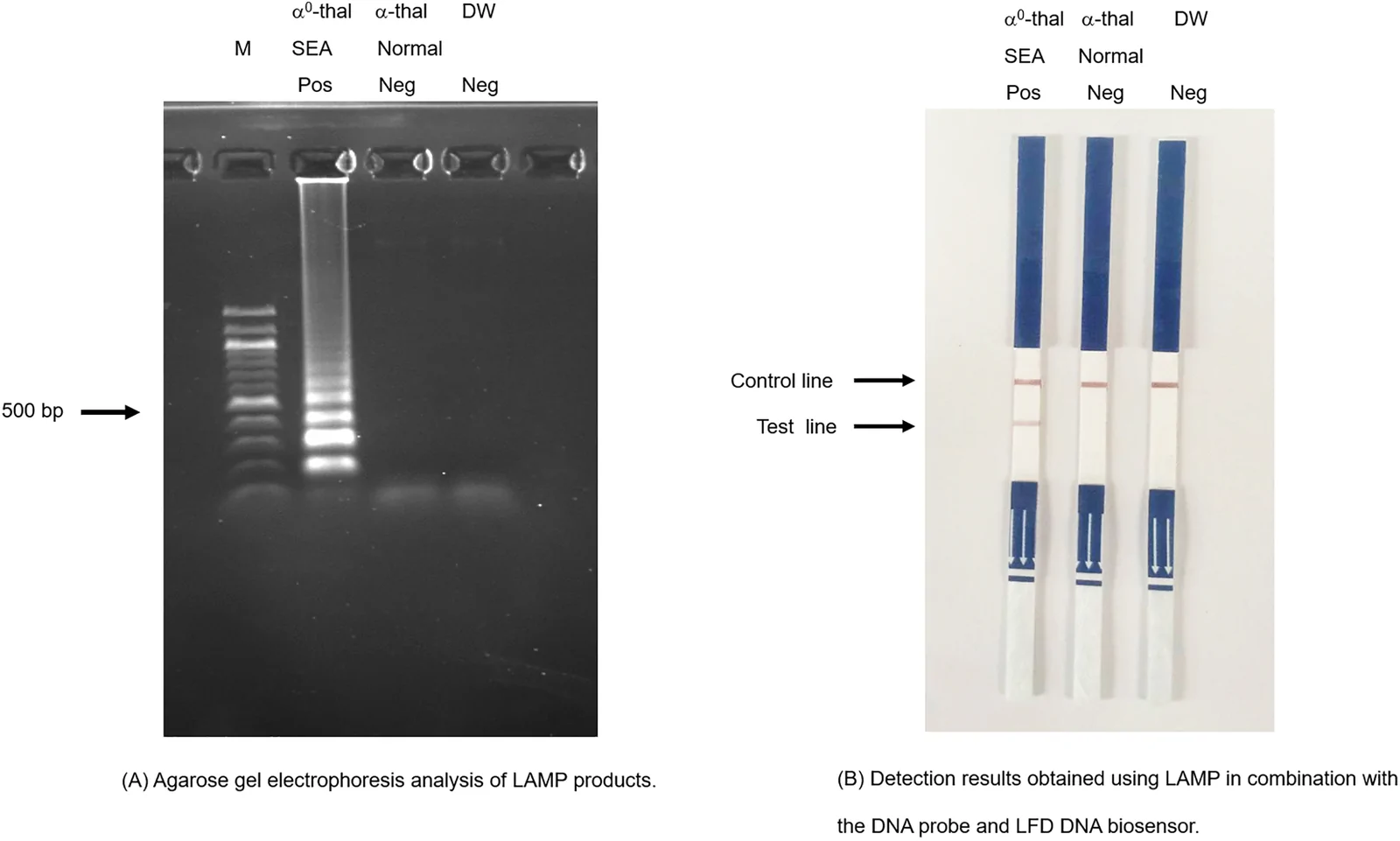
Detection of the α^0^-thalassemia (--SEA deletion) gene using LAMP assay combined with a DNA probe and LFD DNA biosensor. (A) Agarose gel electrophoresis analysis of LAMP products. (B) Detection results obtained using LAMP in combination with the DNA probe and LFD DNA biosensor. Lane M: DNA ladder marker; lane 1: α^0^-thalassemia (--SEA deletion)-positive sample; lane 2: α-thalassemia-negative (normal) sample; lane 3: distilled water (DW) used as a negative control.

### Sensitivity of the one-step LAMP-LFD DNA biosensor

The analytical sensitivity of the one-step LAMP with an LFD DNA biosensor was assessed using 10-fold serial dilutions of α^0^-thalassemia (SEA deletion) genomic DNA, ranging from 20 ng to 20 fg per reaction. The assay successfully detected target DNA down to 200 pg, which was defined as the limit of detection (Fig 5). No signal was observed below this concentration, indicating high analytical sensitivity.

**Fig 5:**
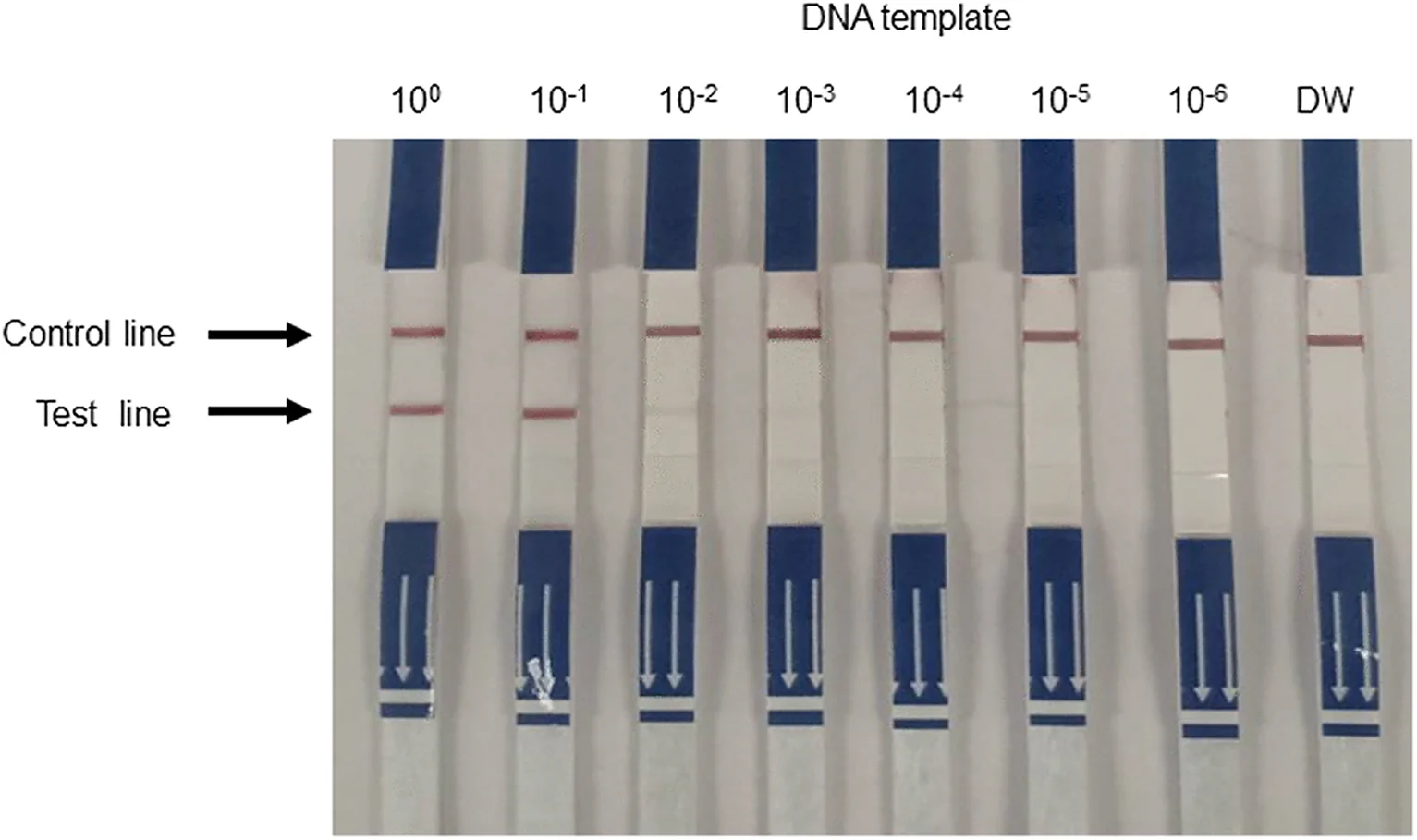
Detection sensitivity of α^0^-thalassemia (SEA deletion) genomic DNA using LAMP combined with a DNA probe and an LFD DNA biosensor. Serial dilutions of the template DNA ranging from 20 ng to 20 fg were tested (initial concentration: 20 ng). The results demonstrated that the minimum detectable concentration for the α^0^-thalassemia (SEA deletion) gene was 200 pg.

### Specificity of the one-step LAMP-LFD DNA biosensor

The specificity of the one-step LAMP-LFD assay was evaluated by testing 20 ng each of α^0^-thalassemia (SEA deletion) genomic DNA and other α0-thalassemia variants, including α^0^-thalassemia (THAI deletion) type. The results demonstrated that no cross-reactive signals were observed with any of the nontarget templates (Fig 6).

**Fig 6:**
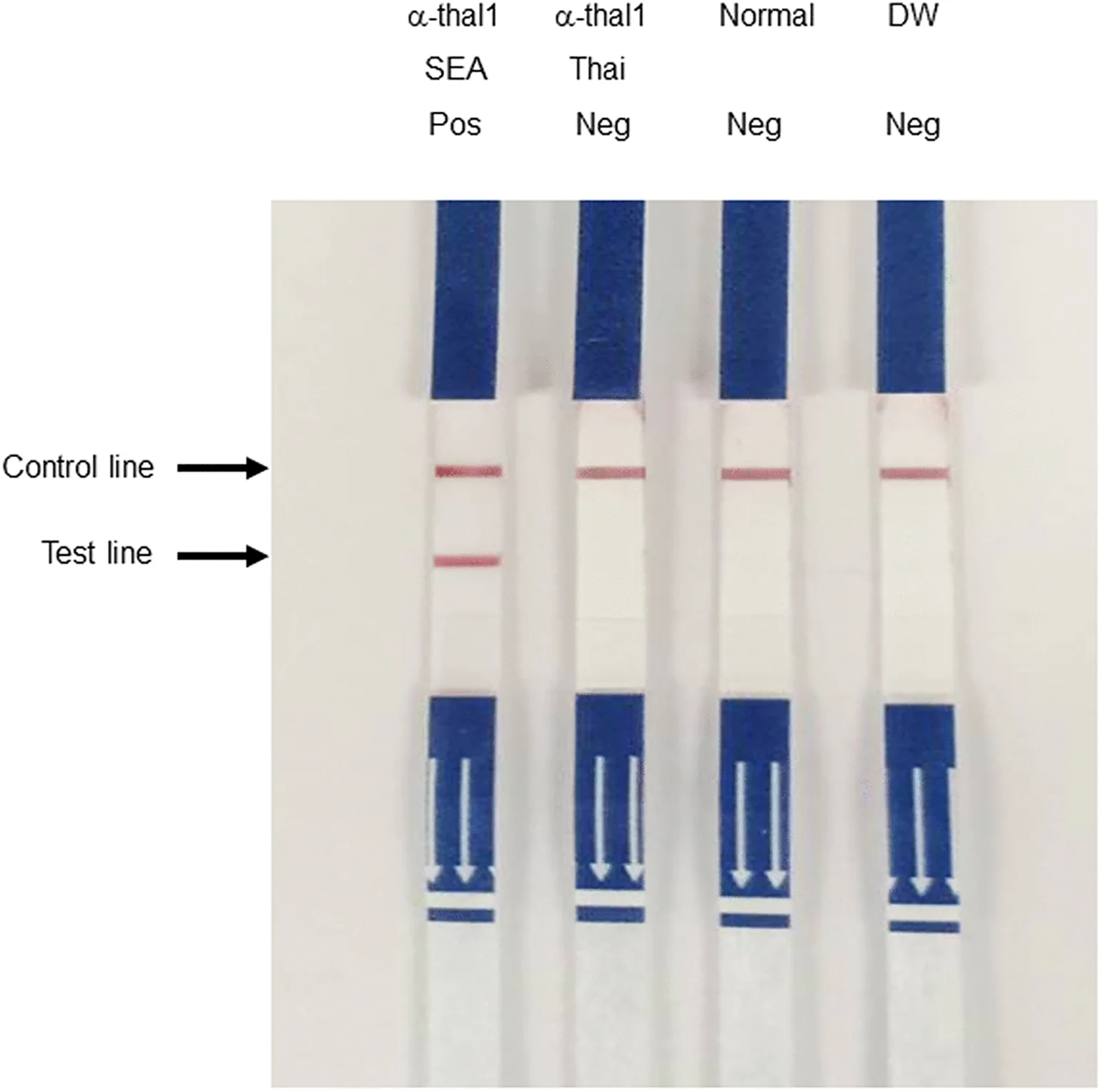
Specificity of the one-step LAMP-LFD assay for α^0^-thalassemia (SEA deletion) type using 20 ng each of DNA template. Lanes 1–4 represent α^0^-thalassemia (SEA deletion)-positive sample, α^0^-thalassemia (THAI deletion) sample, α-thalassemia (SEA deletion)-negative (normal) sample, and distilled water (DW, no-template control), respectively. No cross-hybridization was observed with the one-step LAMP-LFD assay.

### Testing of clinical samples

A total of 96 clinical DNA samples confirmed by conventional PCR were analyzed by LAMP amplification, followed by LFD detection. The results were subsequently compared with those from conventional PCR. The one-step LAMP-LFD assay demonstrated 100% sensitivity and 100% specificity for the diagnosis of α^0^-thalassemia (SEA deletion) type in clinical samples. The one‑step LAMP‑LFD assay demonstrated complete concordance with conventional PCR. All diagnostic performance metrics—including sensitivity, specificity, predictive values, and overall accuracy—reached 100% (95% CI: 92.6%–100%). This absolute agreement was confirmed by a Cohen’s kappa coefficient of 1.0, backed by a highly significant statistical association (Fisher’s exact test, p < 0.0001) (Fig 7 and Tables 2 and 3).

**Fig 7:**
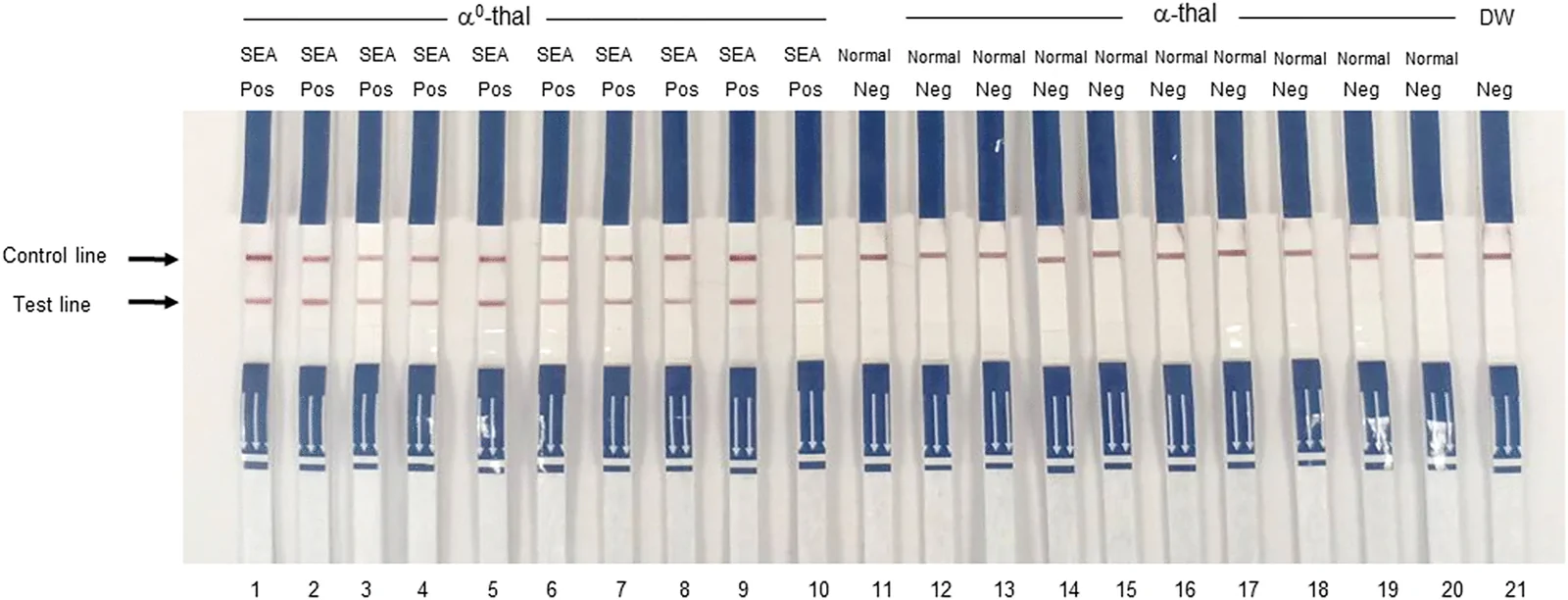
Detection of α^0^-thalassemia (SEA deletion) genomic DNA using one-step LAMP-LFD assay. Lanes 1–10: α^0^-thalassemia (SEA deletion)-positive samples; lanes 11–20: α^0^-thalassemia (SEA deletion)-negative (normal) samples; lane 21: distilled water (DW) used as a negative control.

**Table 2:** Comparison of one-step LAMP-LFD and conventional PCR results for the identification of clinical samples carrying the α^0^-thalassemia (SEA deletion) gene.

One-Step LAMP-LFD assay	α^0^-thalassemia of SEA deletion type results by PCR		Total
	Positive	Negative	
**Positive**	48	0	48
**Negative**	0	48	48
**Total**	48	48	96

**Table 3:** Statistical performance parameters of the one-step LAMP-LFD assay compared with conventional PCR as the reference standard*.

Parameter	Value	95% Confidence Interval (CI)
Sensitivity	100.00%	92.59% to 100.00%
Specificity	100.00%	92.59% to 100.00%
Positive Predictive Value (PPV)	100.00%	92.59% to 100.00%
Negative Predictive Value (NPV)	100.00%	92.59% to 100.00%
Overall Accuracy	100.00%	92.59% to 100.00%
Cohen’s kappa coefficient (κ)	1.000	1.000 to 1.000

* There was a statistically significant association between the LAMP-LFD assay and conventional PCR (Fisher’s exact test, *p* < 0.0001).

## Discussion

The LAMP reaction was performed at 61°C for 60 min, which was notably faster than conventional PCR (typically requiring 2–3 h to complete). The LAMP assay requires no special instruments, and the results can be reported within 1 h, excluding DNA preparation time. The one-step LAMP-LFD assay was faster than LAMP with gel electrophoresis (2 h and 30 min) and PCR with gel electrophoresis (3 h and 30 min). These factors limit the widespread use of PCR in α^0^-thalassemia screening. Besides its convenience, the one-step LAMP-LFD method simplifies hybridization, provides easy-to-read results, and reduces the need for foreign technology imports. The LAMP technique based on the LFD assay has been successfully applied for the detection of Mycobacterium tuberculosis [23] and shrimp Taura syndrome virus [24].

In the present study, the novel one-step LAMP-LFD technique could be used to detect α^0^-thalassemia (SEA deletion) type in clinical samples. These results were in accordance with previous PCR results. Therefore, negative one-step LAMP-LFD test results could be used to exclude α^0^-thalassemia (SEA deletion) carriers. In addition, the one-step LAMP-LFD DNA biosensor assay demonstrated high specificity and sensitivity for the identification of α^0^-thalassemia (SEA deletion) traits (Tables 2 and 3), with a detectable range of 200 pg to 20 ng (Fig 5).

Furthermore, the one-step LAMP-LFD assay confirmed the identity of the specific α^0^-thalassemia (SEA deletion) amplicon and avoided the use of carcinogens, including ethidium bromide. The test platform can be easily adapted for the rapid detection of other α^0^-thalassemia types, including the THAI deletion, simply by designing appropriate sets of LAMP primers and specific FITC probes compatible with the generic one-step LFD system employed. Since the cost of the one-step LAMP-LFD assay is comparable to that of standard PCR followed by gel electrophoresis, it represents a highly sensitive, safe, and rapid alternative for the detection of α^0^-thalassemia (SEA deletion). Therefore, this technique could be applicable for detecting minimal amounts of α^0^-thalassemia (SEA deletion) in clinical specimens. Regarding specificity, no cross-reactivity with other α^0^-thalassemia types, including the THAI deletion, was observed (Fig 6).

LAMP has been recognized as a rapid approach for thalassemia genotyping. Jomoui et al. developed a phenol red–based colorimetric LAMP assay to detect α^0^-thalassemia deletions (--SEA and --THAI) [14]. While closed-tube colorimetric formats help minimize carryover contamination, the phenol red system has notable drawbacks. Because it relies on subtle pH shifts, transitional hues such as orange can introduce subjectivity, leaving results open to misinterpretation.

By contrast, our LAMP-LFD assay delivers an unambiguous readout: a distinct band on the strip generated through target-specific probe hybridization. This eliminates the uncertainty inherent in color-based detection. Because the reaction tubes must be opened prior to strip analysis, carryover contamination remains a critical hurdle for LAMP-LFD assays. We suggested that separation between amplification and detection zones, used aerosol-barrier pipette tips to eliminate carryover DNA. These combined safeguards protect the assay from false positives, keeping the final data clear and dependable. Compared with other LAMP assays—such as those using metal-ion indicators or fluorescence—our platform resolves several diagnostic and visualization challenges. Metal-ion indicators like hydroxy naphthol blue or calcein are inexpensive but prone to subjective color changes under ambient light, lack multiplexing capability, and risk false positives from non-specific byproducts or Mg^2^ ^+^ depletion [26]. Fluorescence-based assays, meanwhile, require specialized readers or UV excitation sources, and intercalating dyes often bind non-specifically to primer-dimers. Multiplexing remains limited without complex fluorophore systems [27].

At roughly $9 USD per test, our lateral flow strips are cost-comparable to the phenol red assay reported by Jomoui et al. [14]. However, our system avoids the need for PCR machines or optical readers, lowering equipment barriers. More importantly, the LAMP-LFD format replaces subjective color calls with a clear binary outcome—positive or negative—reducing the expense of retesting ambiguous samples. The slight increase in strip cost is outweighed by improved diagnostic accuracy and strong applicability in resource-limited settings. This study has several limitations that should be acknowledged. First, the study cohort of 96 subjects (48 positive/48 negative) represents a balanced sample. However, a larger sample size should be pursued, particularly including heterozygous carriers, to further validate the assay’s reliability across more diverse population groups. Second, the current assay was designed exclusively for SEA deletion. The development of a multiplex LAMP-LFD capable of simultaneously detecting SEA and THAI deletions should be considered to broaden the clinical utility of this approach. Third, the assay should be evaluated using alternative specimen types, including dried blood spots or buccal swabs, to enhance its flexibility for point-of-care testing applications, particularly in resource-limited or field settings. Fourth, the effect of potential inhibitors commonly encountered in clinical samples, including heme and heparin, should be assessed to confirm the robustness and reliability of the assay under real-world conditions. In addition, our current LAMP-LFD assay does not detect the THAI deletion. This confirms our primers target only the SEA deletion, despite both being α ^0^-thalassemia mutations. This design aligns with local epidemiological data: the SEA deletion causes the vast majority of local cases, while the THAI deletion remains very rare. In future work, we plan to expand this into a multiplex platform using a dual-band strip to detect both SEA and THAI deletions in a single run. Adapting the LFD to feature multiple probe lines on a single strip enables the concurrent detection of distinct α-thalassemia deletions. This capacity for multiplexing proves essential for screening initiatives across Southeast Asia, where clinicians routinely encounter a wide variety of mutation profiles.

## Conclusion

In this study, a one-step LAMP-LFD method was developed for the identification of α^0^-thalassemia (SEA deletion) type. The assay is simple and can be rapidly performed, and the results can be read by the naked eye. Moreover, the assay shows strong potential as a cost-effective routine screening tool for α^0^-thalassemia (SEA deletion) type in hospital or medical laboratory settings. In high-prevalence countries, particularly Thailand and other populations across Southeast Asia, this novel one-step LAMP-LFD DNA biosensor can serve as a useful tool for ruling out α^0^-thalassemia, especially the SEA deletion type, to prevent and control severe thalassemia.

## Supporting information

S1 File Raw images. (PDF)
