# Supplementary material for: Development of loop-mediated isothermal amplification (LAMP) combined with a lateral flow dipstick DNA biosensor for detection of α thalassemia 1 [Southeast Asia (SEA), --SEA deletion]
Source: PLoS One. 2026 Oct 12;21(10):e0360555. doi: 10.1371/journal.pone.0360555 (PMC13657280; doi:10.1371/journal.pone.0360555)

<u>Supporting Information files (S1)</u>

Original gel pictures and images used in Figures 1-7

(uncropped and unadjusted)

Fig1. Nucleotide sequence of the α thalassemia breakpoint region gene specific for α0 -thalassemia, SEA deletion genome (GenBank: KX458114.1). The positions and orientations of the LAMP primer set comprising F3 (light blue), B3 (light blue), FIP (F1c (pink) -TTT-F2 (green), biotinylated BIP (B1c (pink) -TTT-B2 (green), and the FITC-labeled LB probe (red) are indicated by arrows and highlighted sequences.

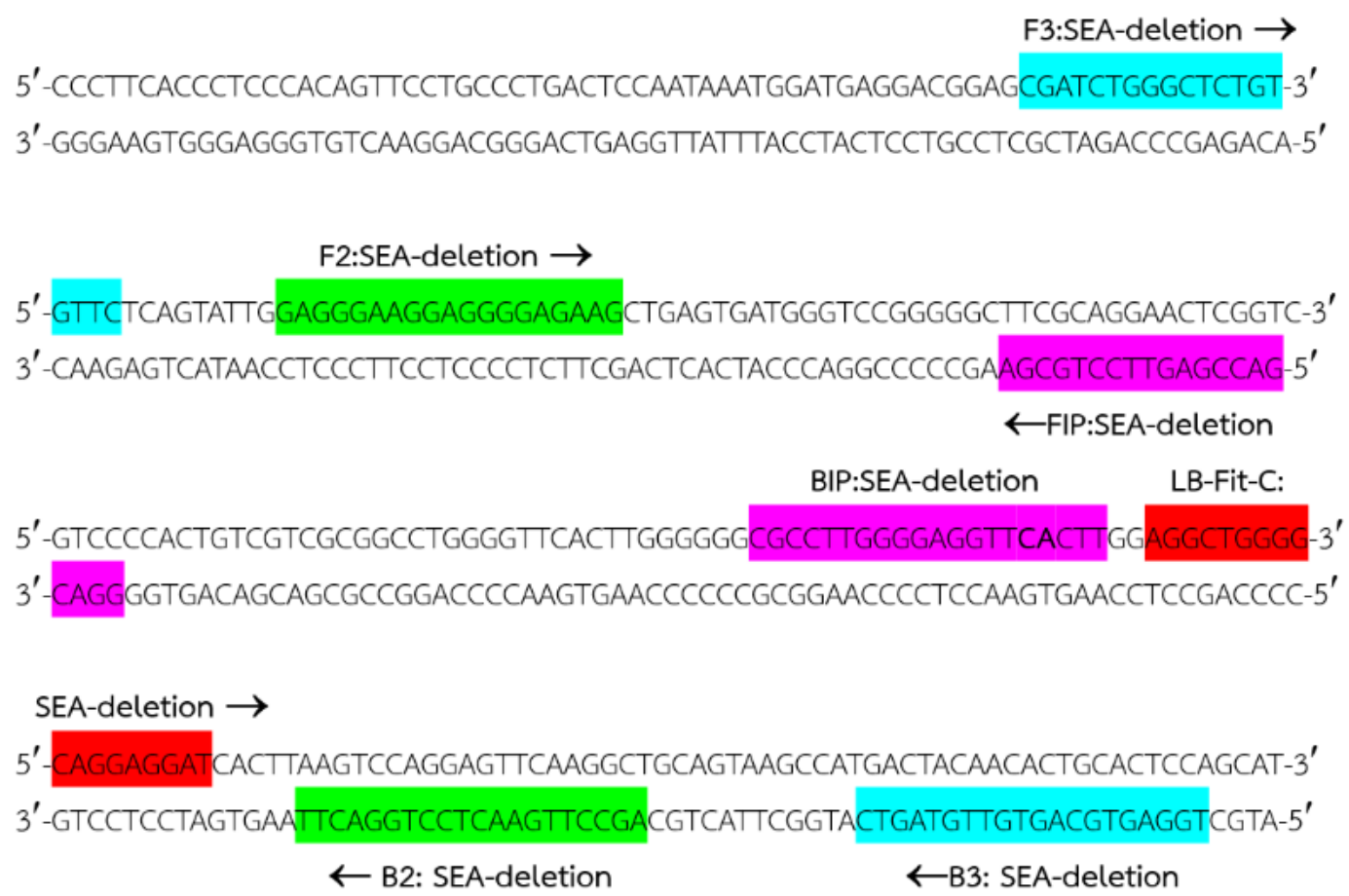

Fig 2. Optimization of LAMP reaction conditions. (A) Optimization of the reaction temperature. Lane 1 (M) represents the DNA ladder marker; lanes 2–5 show LAMP reactions performed at 59°C, 61°C, 63°C, and 65°C, respectively; and lane 6 (Neg) represents the negative control. All LAMP reaction products were analyzed by 1.5% agarose gel electrophoresis. (B) Optimal MgSO4 concentration assay. Lane 1 (M) represents the DNA ladder marker; lanes 2–5 show LAMP reactions performed at 2, 4, 6, 8, and 10 mM MgSO4 for 60 min; lane 6 (Pos) presents the positive control; and lane 7 (Neg) represents the negative control. All LAMP reaction products were analyzed by 1.5% agarose gel electrophoresis. (C) Optimal dNTP concentration. Lane 1 (M) represents the DNA ladder marker; lanes 2–5 show LAMP reactions performed at 0.4, 0.8, 1.2, and 1.6 mM dNTP for 60 min; lane 6 (Pos) presents the positive control; and lane 7 (Neg) represents the negative control. All LAMP reaction products were analyzed by 1.5% agarose gel electrophoresis.

(2A) Optimization of the reaction temperature

Lane 4–9 (from left to right): M; 59 °C; 61 °C; 63 °C; 65 °C; and Negative control.

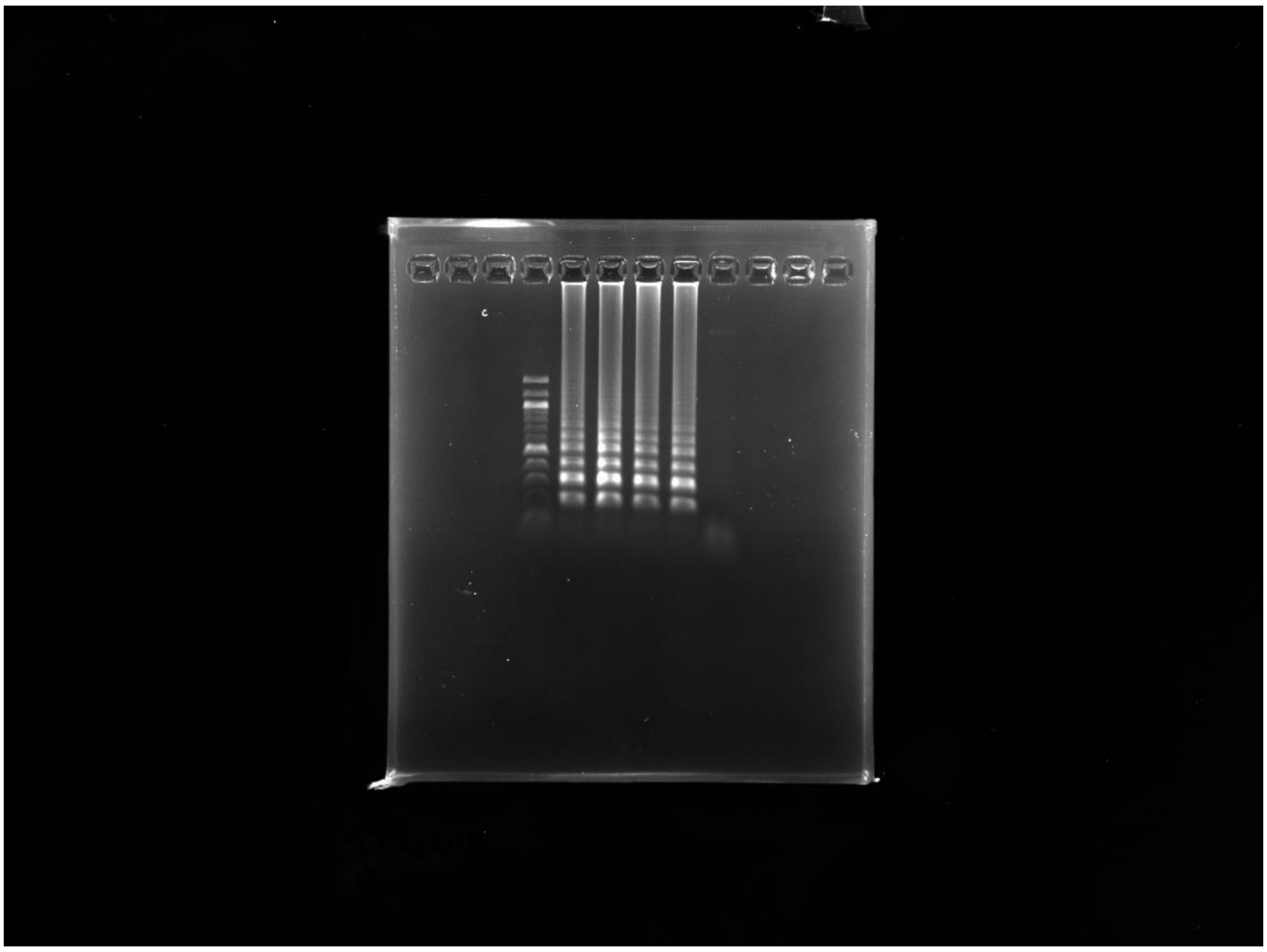

(2B) Optimal $MgSO_4$ concentration

Lane 9-15(from left to right): Land 9 (M) represents the DNA ladder marker, lanes 10–13 show LAMP reactions performed at 2, 4, 6, 8, and 10 mM $MgSO_4$ for 60 min; lane 14 (Pos) presents the positive control; and lane 15 (Neg) represents the negative control.

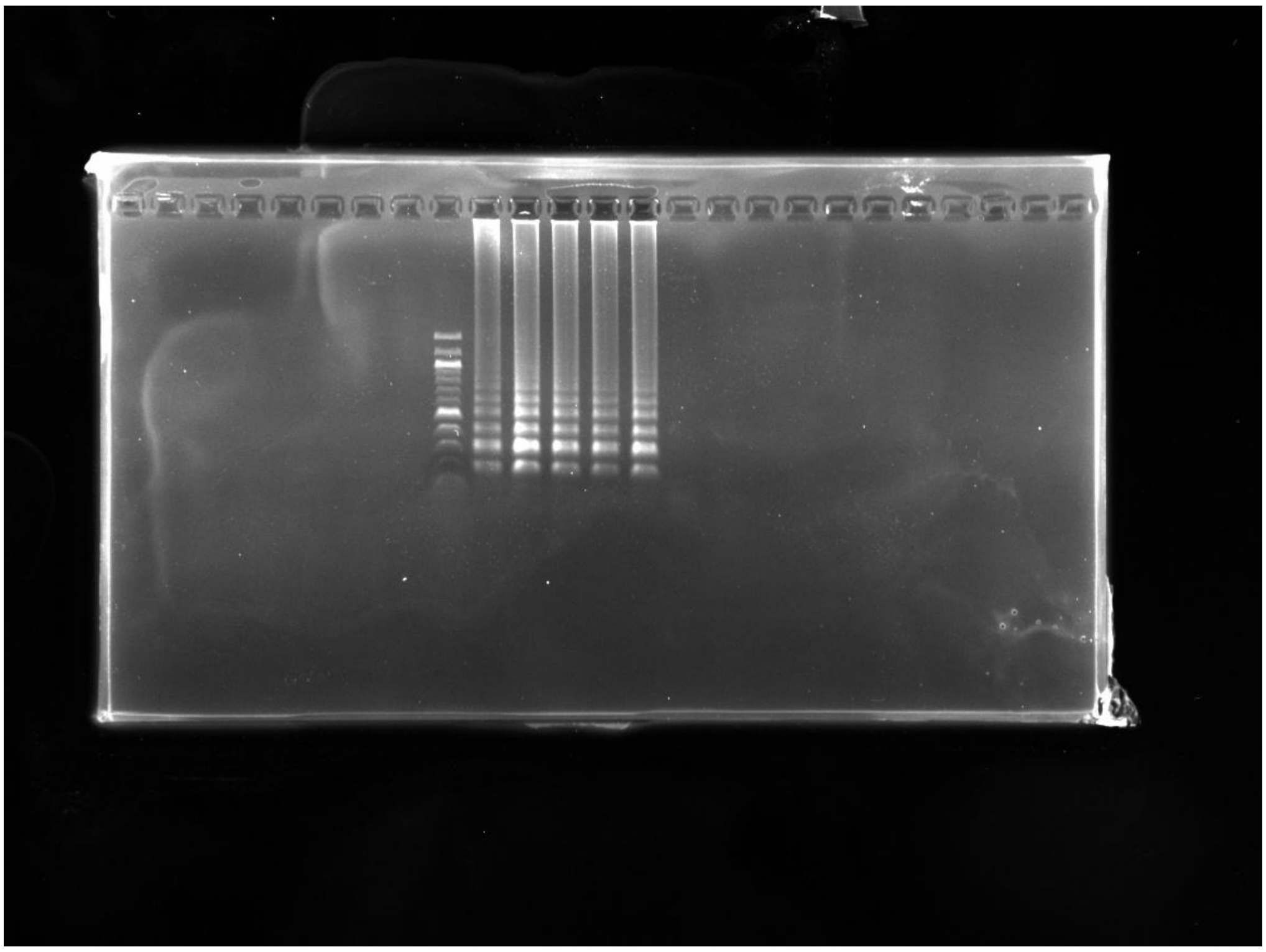

(2C) Optimal dNTP concentration

Lane 9-15(from left to right): lane 9 (M) represents the DNA ladder marker; lanes 10–13 show LAMP reactions performed at 0.4, 0.8, 1.2, and 1.6 mM dNTP for 60 min; lane 14 (Pos) presents the positive control; and lane 15 (Neg) represents the negative control.

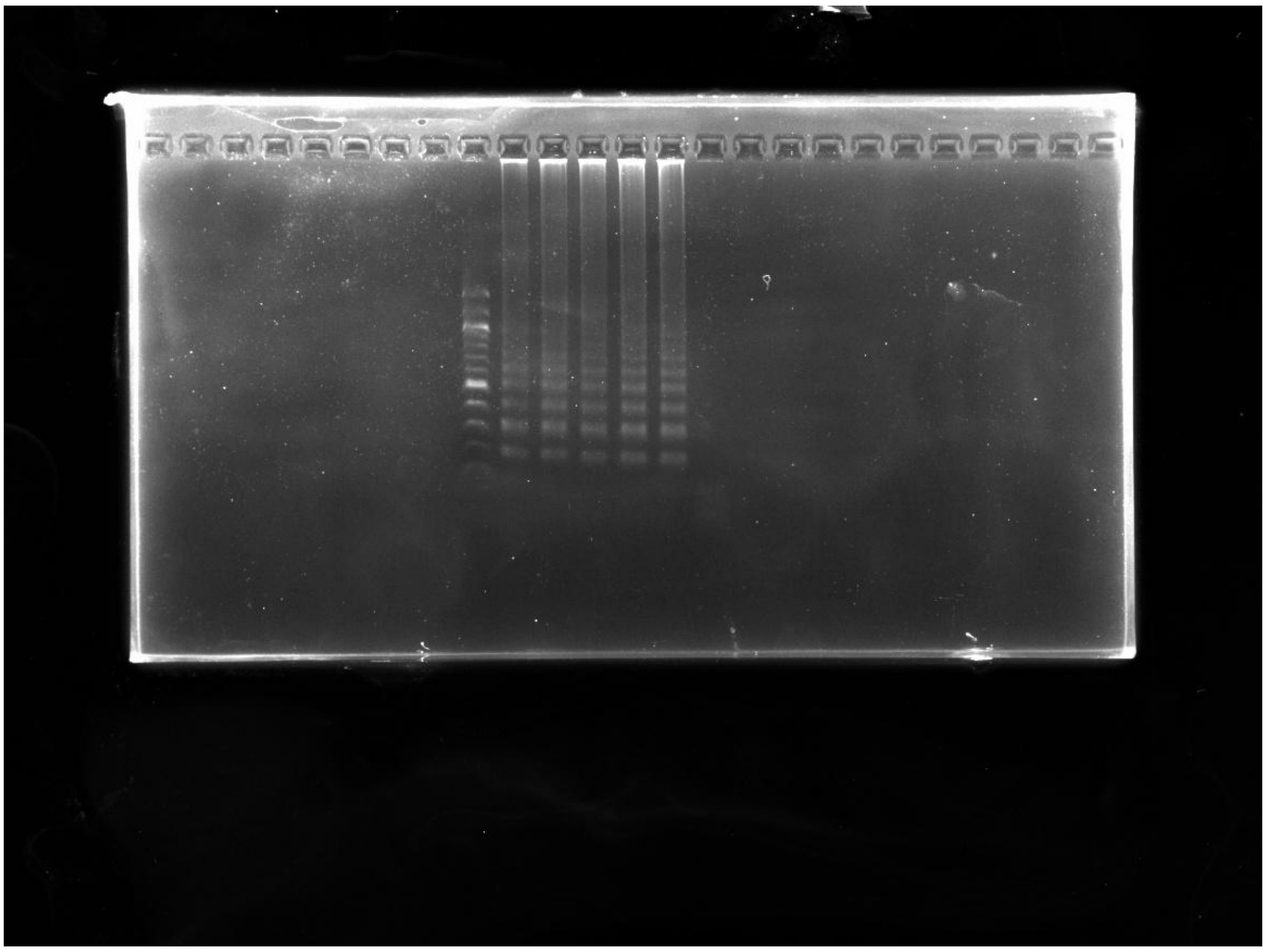

Fig 3. Optimum FITC-labeled DNA probe**.** Results of LAMP combined with a DNA probe and LFD DNA biosensor at different FITC-labeled DNA probe concentrations (0.1, 0.2, 0.3, and 0.4 μM) in the one-step LAMP-LFD reaction.

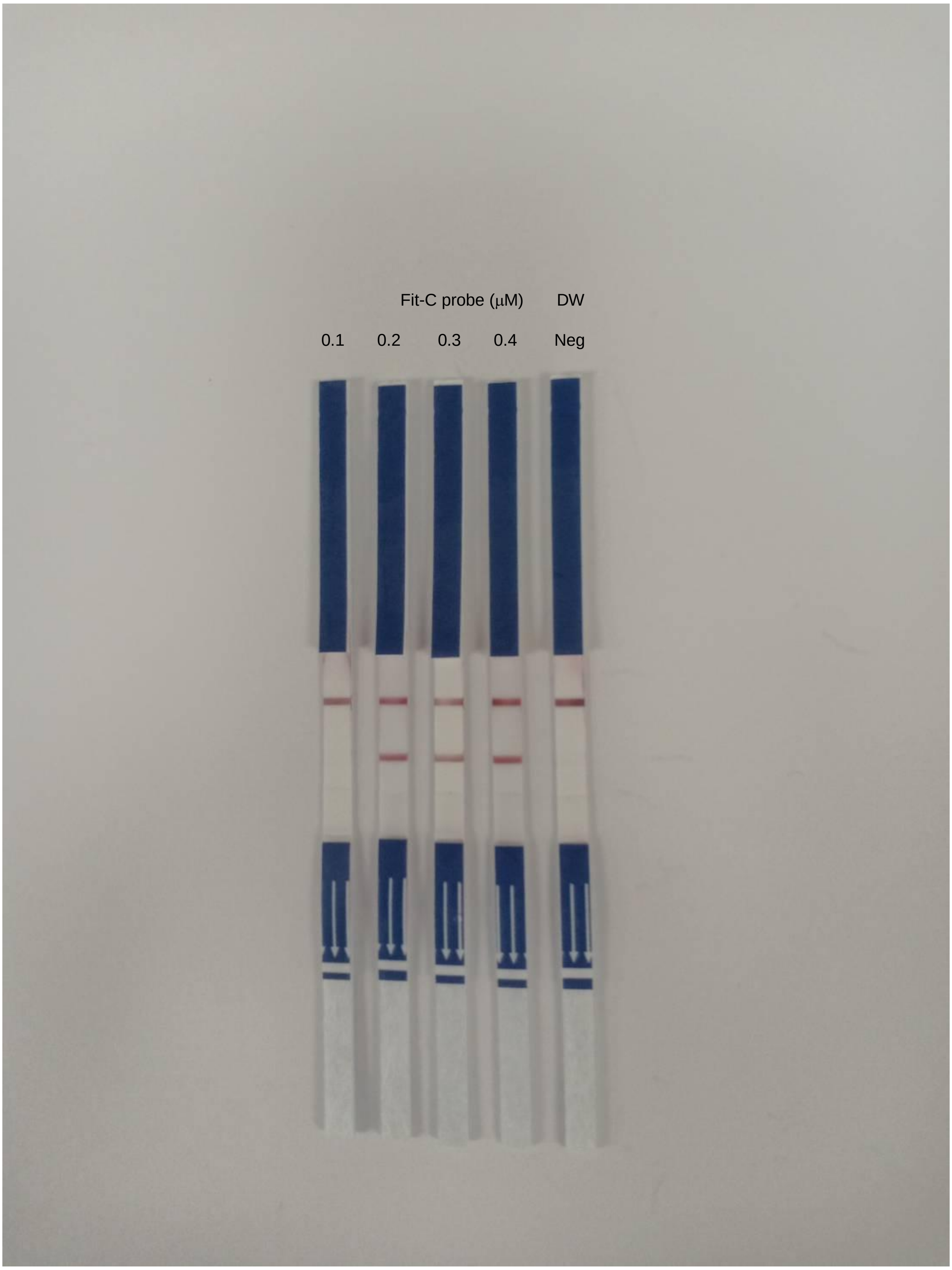

Fig 4. Detection of the α⁰-thalassemia (--SEA deletion) gene using LAMP assay combined with a DNA probe and LFD DNA biosensor. (A) Agarose gel electrophoresis analysis of LAMP products. (B) Detection results obtained using LAMP in combination with the DNA probe and LFD DNA biosensor. Lane M: DNA ladder marker; lane 1: α^0-thalassemia (--SEA deletion)-positive sample; lane 2: α-thalassemia-negative (normal) sample; lane 3: distilled water (DW) used as a negative control.

(4A) Agarose gel electrophoresis analysis of LAMP products

Lane 4-7 (from the left): lane 4 (M) represents the DNA ladder marker, lane 5 α^0-thal SEA positive, lane 6 α^0-thal Normal positive, lane 7 distilled water (DW).

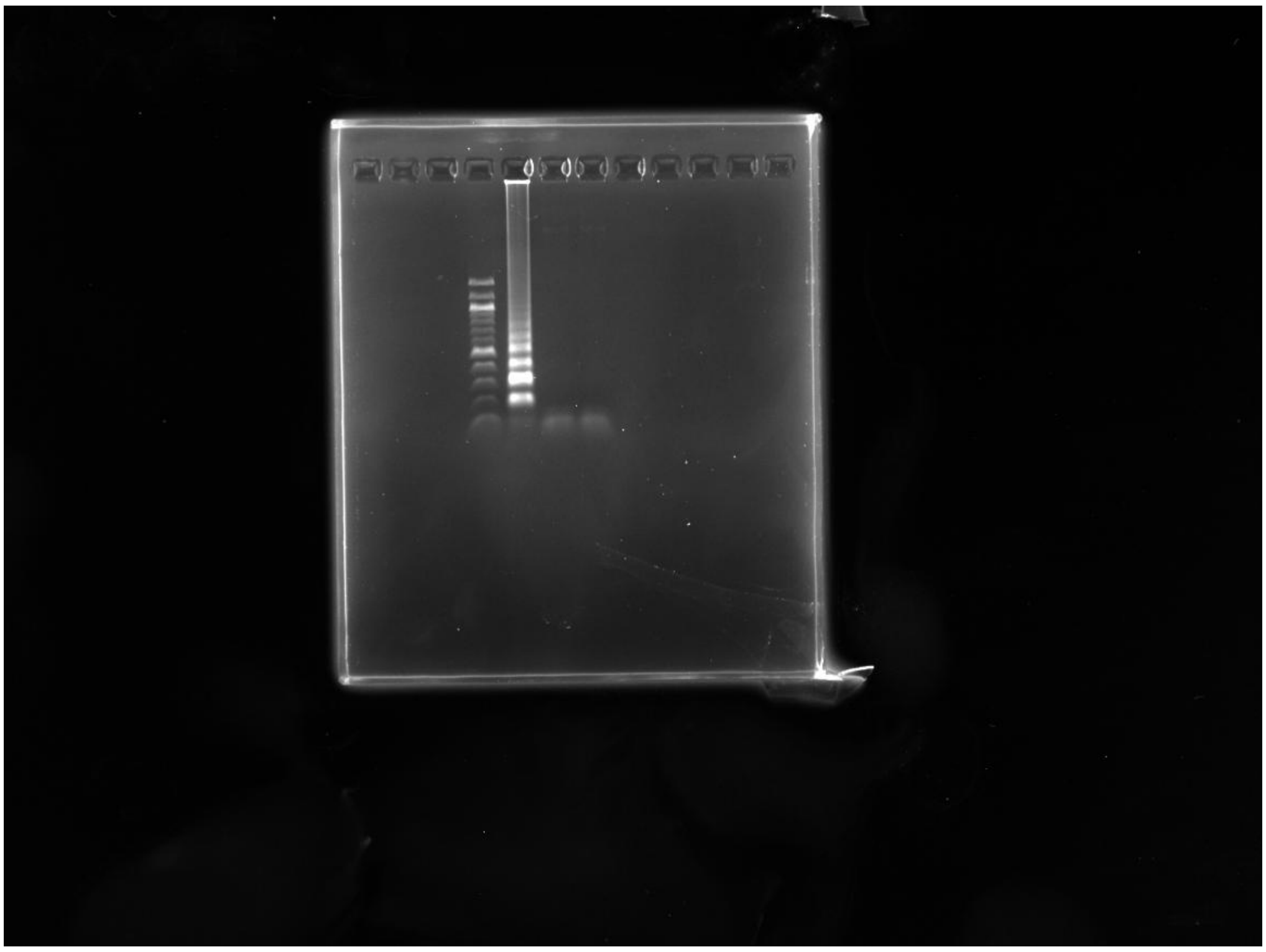

(4B) Detection results obtained using LAMP in combination with the DNA probe and LFD DNA biosensor

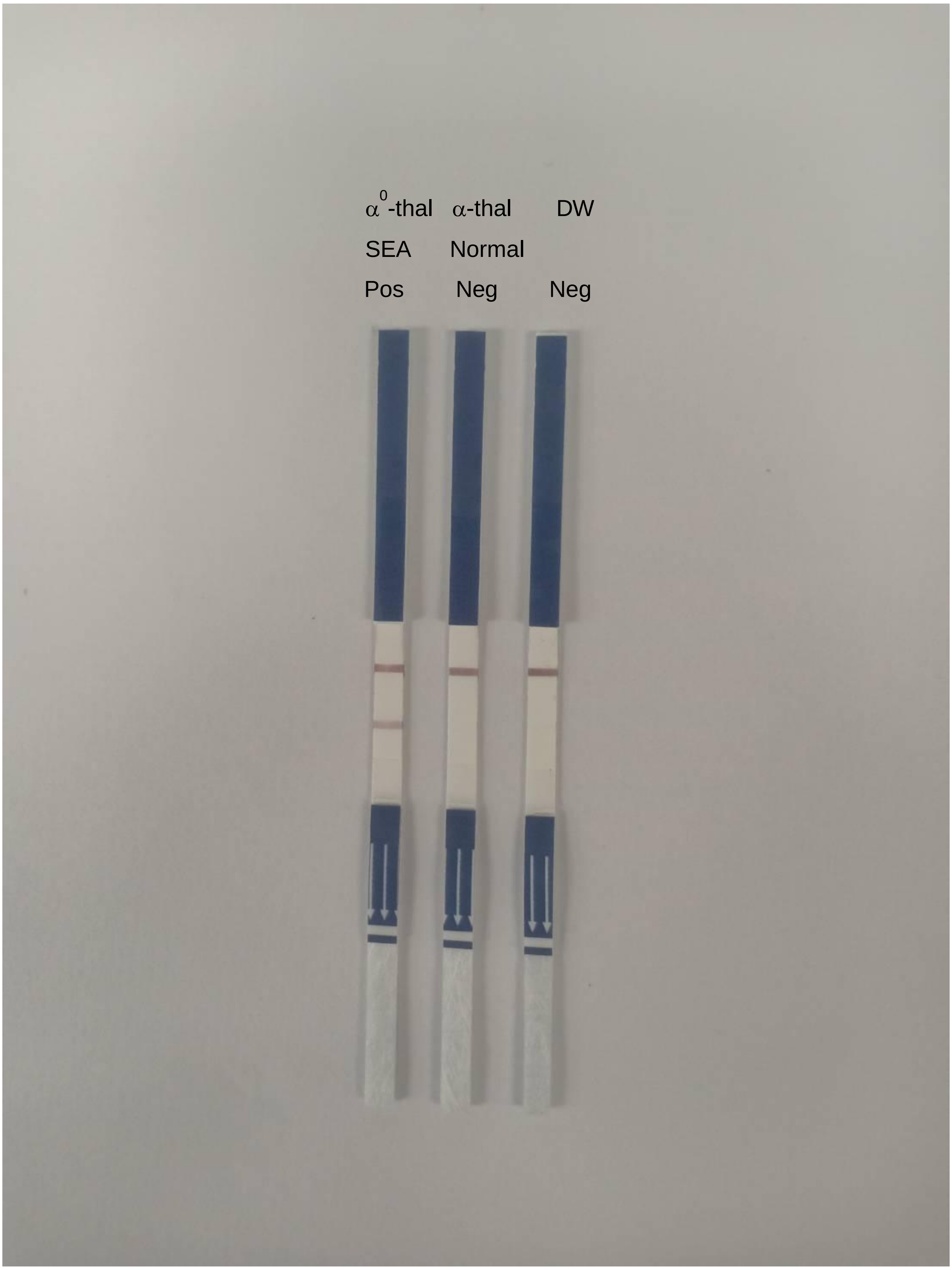

Fig 5. Detection sensitivity of α0-thalassemia (SEA deletion) genomic DNA using LAMP combined with a DNA probe and an LFD DNA biosensor. Serial dilutions of the template DNA ranging from 20 ng to 20 fg were tested (initial concentration: 20 ng). The results demonstrated that the minimum detectable concentration for the α0-thalassemia (SEA deletion) gene was 200 pg.

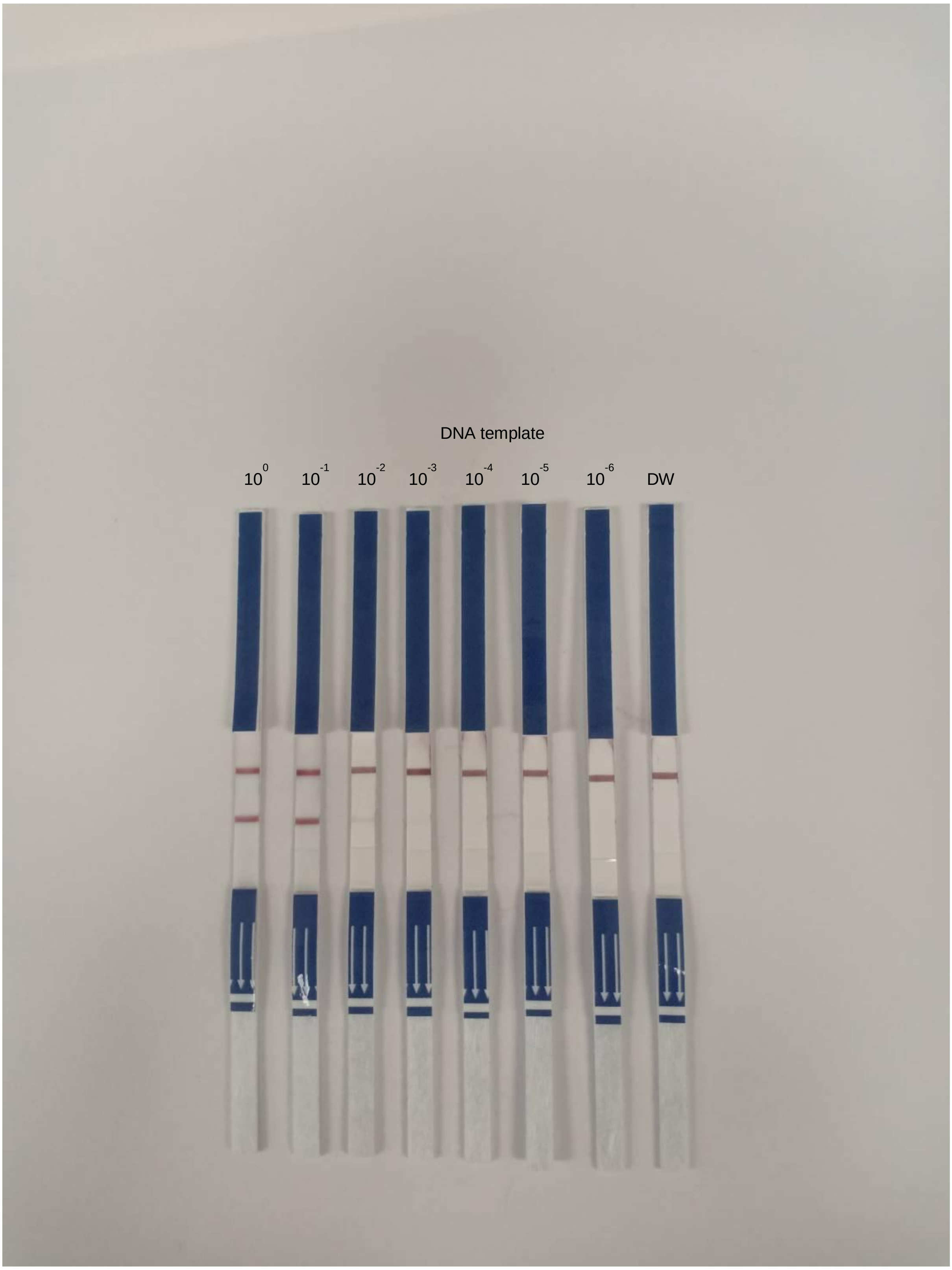
DNA template
10^0 10^-1 10^-2 10^-3 10^-4 10^-5 10^-6 DW

Fig 6. Specificity of the one-step LAMP-LFD assay for α0-thalassemia (SEA deletion) type using 20 ng each of DNA template. Lanes 1–4 represent α0-thalassemia (SEA deletion)-positive sample, α0-thalassemia (THAI deletion) sample, α-thalassemia (SEA deletion)-negative (normal) sample, and distilled water (DW, no-template control), respectively. No cross-hybridization was observed with the one-step LAMP-LFD assay.

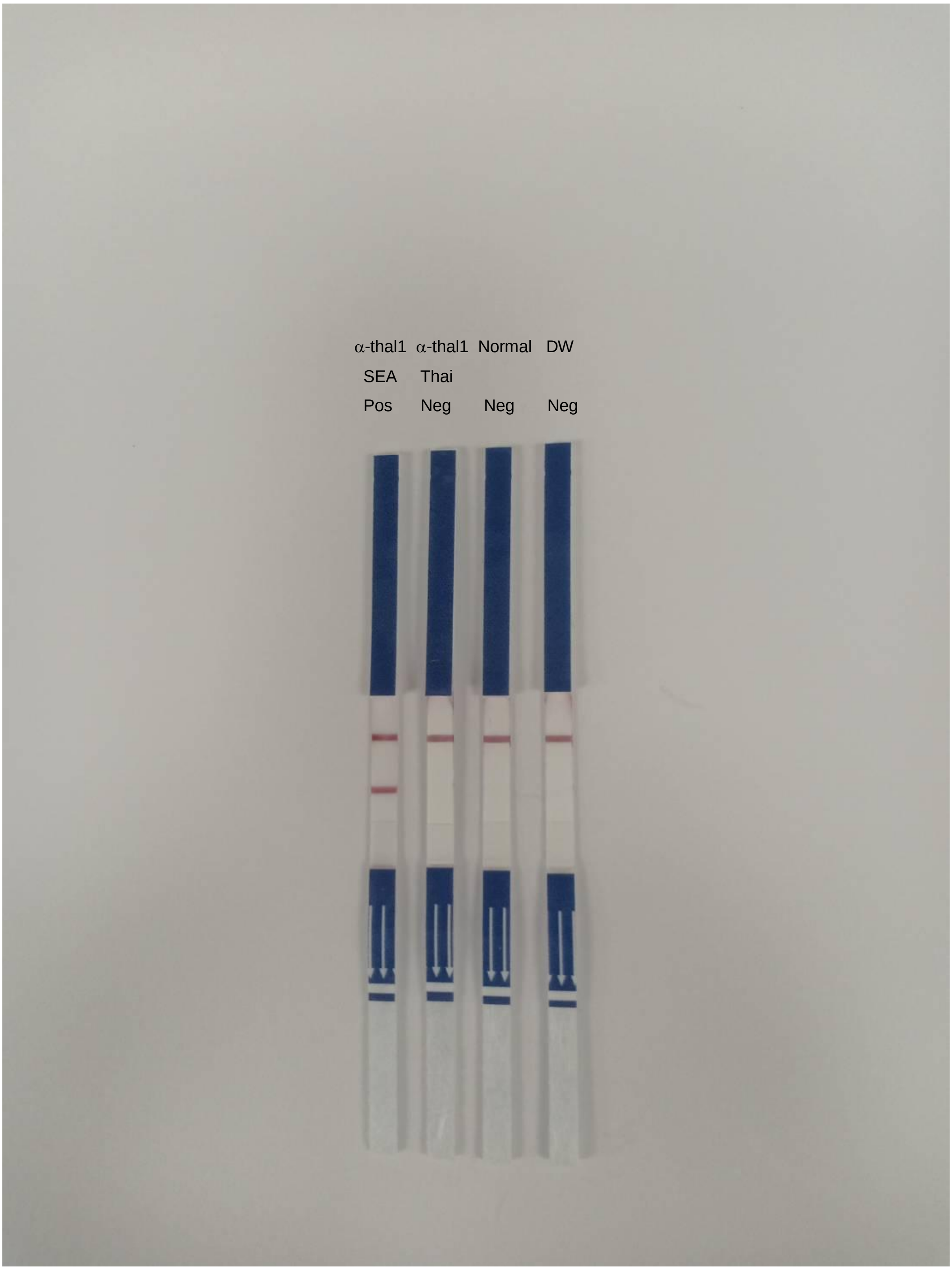
α-thal1 SEA Pos
α-thal1 Thai Neg
Normal Neg
DW Neg

Fig 7. Detection of α^0-thalassemia (SEA deletion) genomic DNA using one-step LAMP-LFD assay. Lanes 1–10: α^0-thalassemia (SEA deletion)-positive samples; lanes 11–20: α0-thalassemia (SEA deletion)-negative (normal) samples; lane 21: distilled water (DW) used as a negative control.

From the left: Lanes 1–10: α^0-thalassemia (SEA deletion)-positive samples; lanes 11–20: α0-thalassemia (SEA deletion)-negative (normal) samples; lane 21: distilled water (DW) used as a negative control.

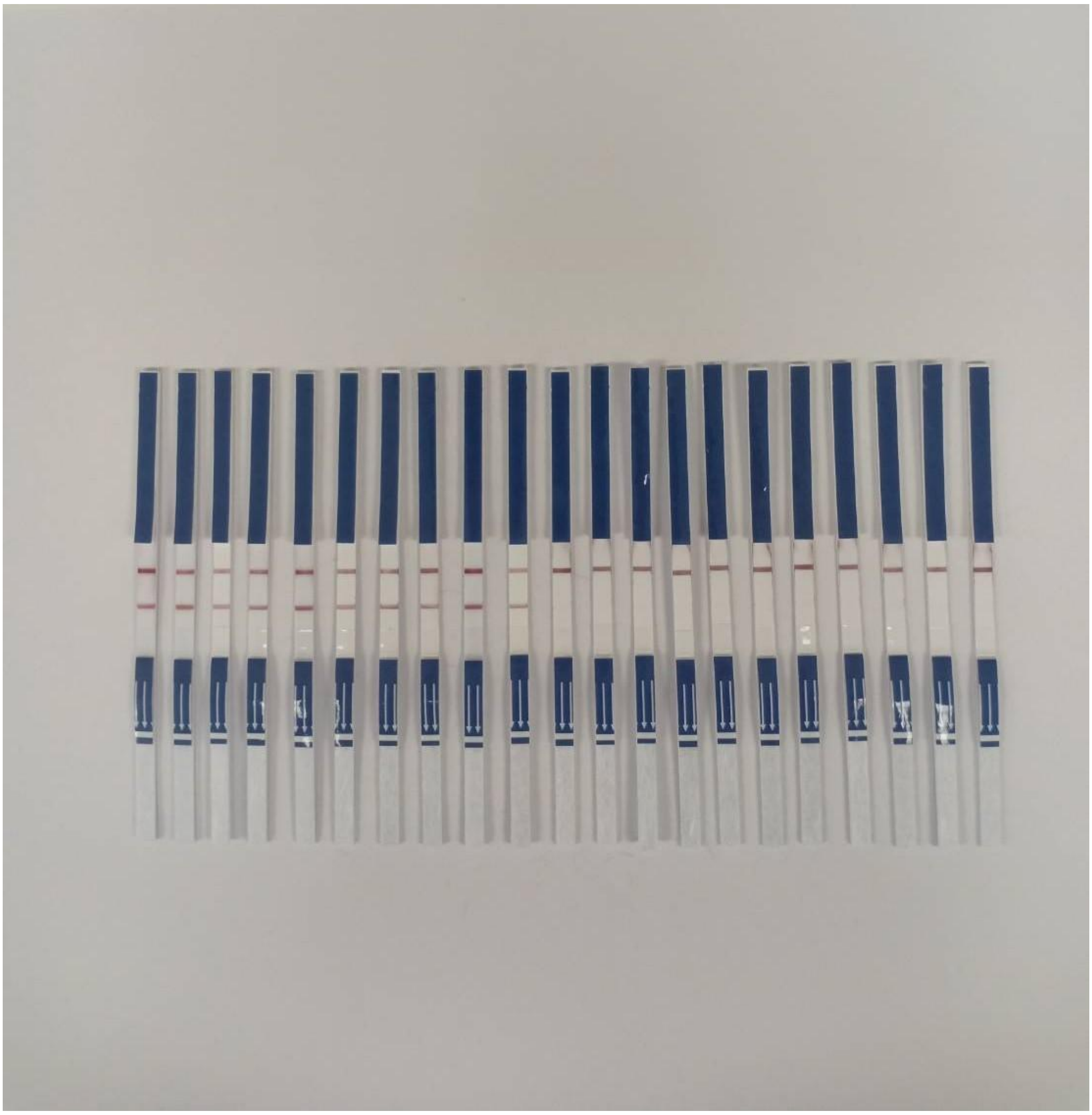

Data from the triplicate evaluation of 96 DNA samples, comprising 48 α^0-thalassemia- positive and 48 α^0-thalassemia- negative samples.

(A) The first replicate (Sample 1-20)

From left to right: Lanes 1–10: SEA-positive samples; Lanes 11–20: SEA-negative samples; Lane 21: distilled water (DW) as a negative control

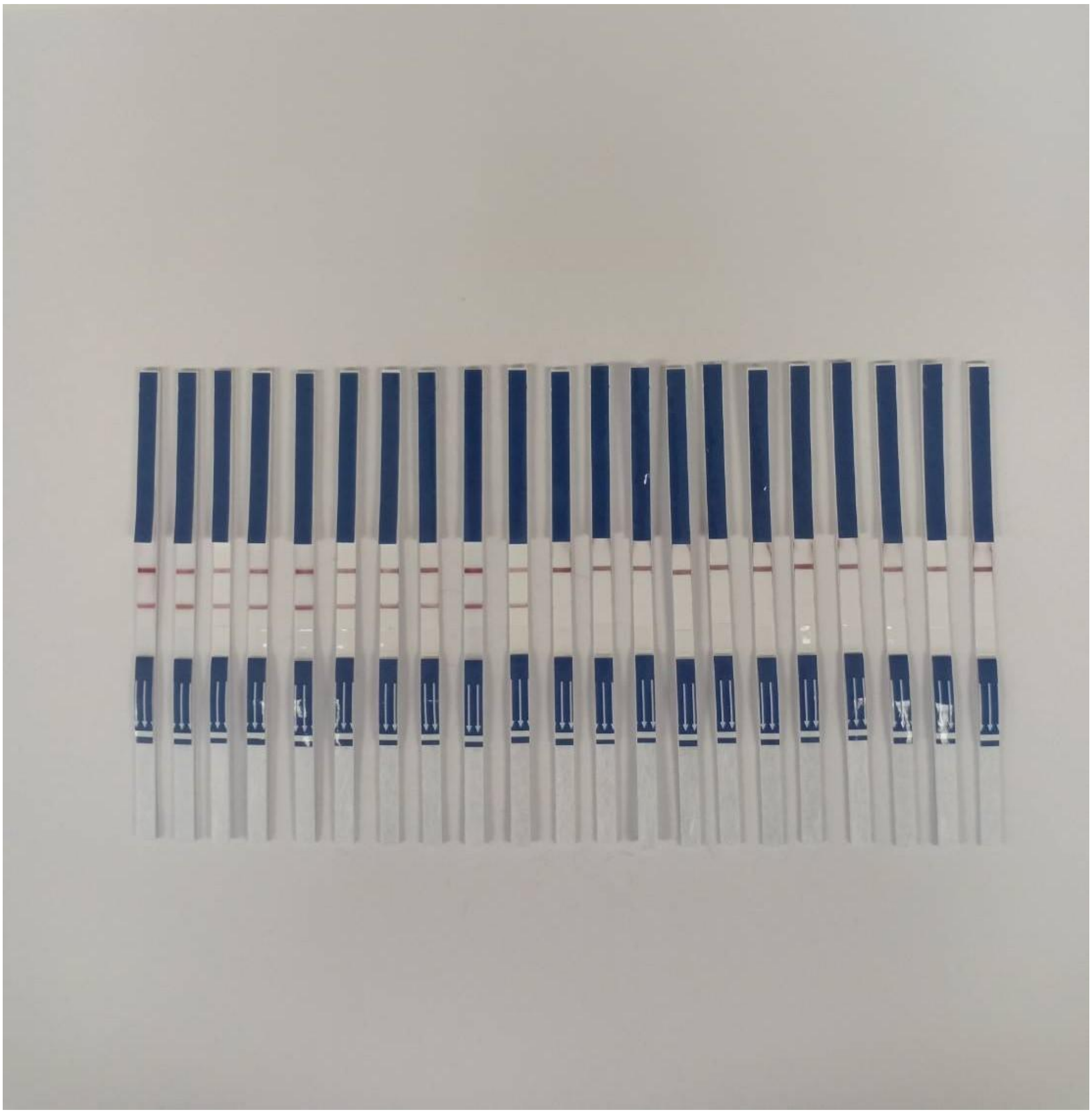

(B) The second replicate (Sample 1-20)

From left to right: Lanes 1–10: SEA-positive samples; Lanes 11–20: SEA-negative samples; Lane 21: distilled water (DW) as a negative control

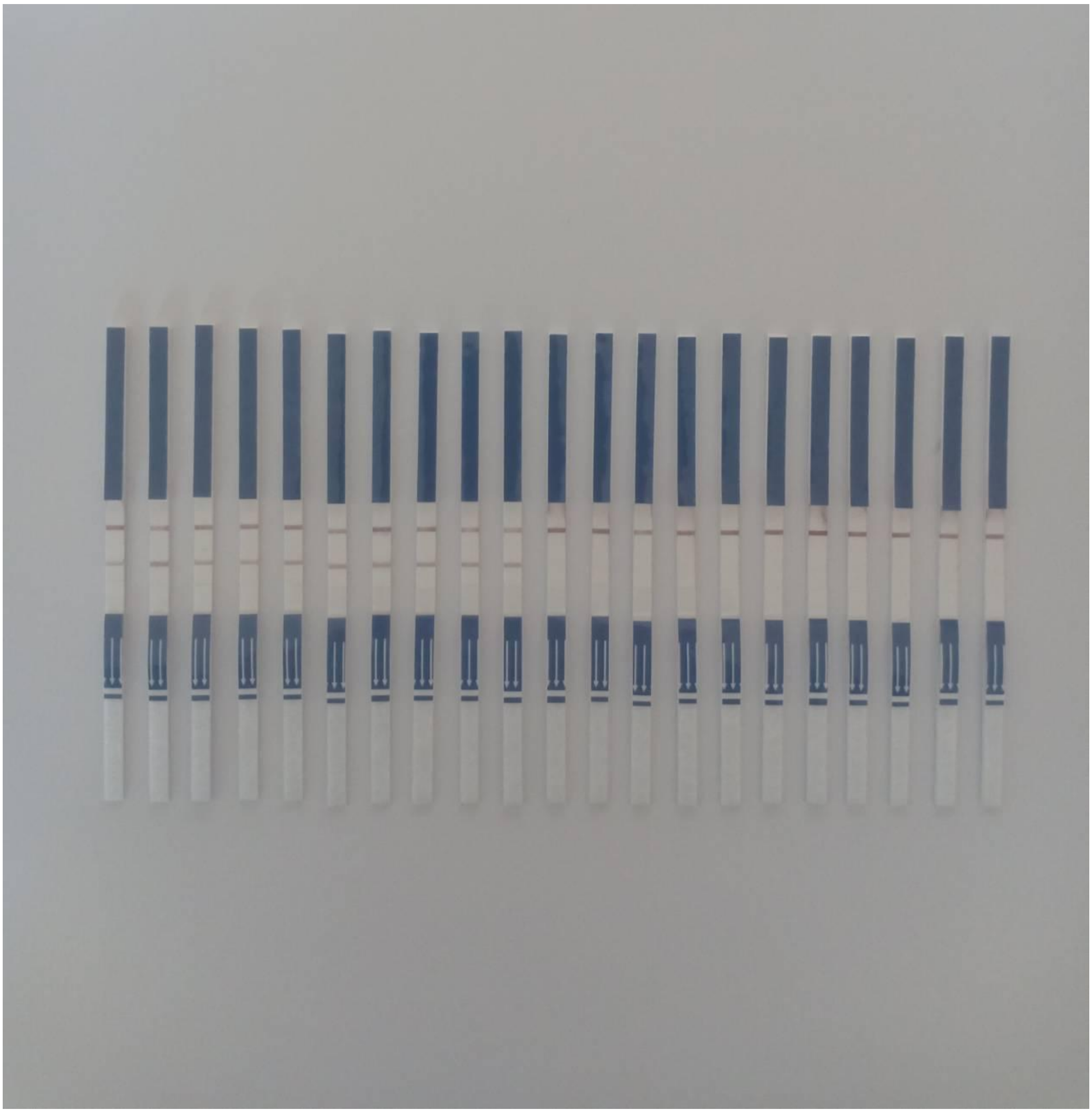

(C) The third replicate (Sample 1-20)

From left to right: Lanes 1–10: SEA-positive samples; Lanes 11–20: SEA-negative samples; Lane 21: distilled water (DW) as a negative control

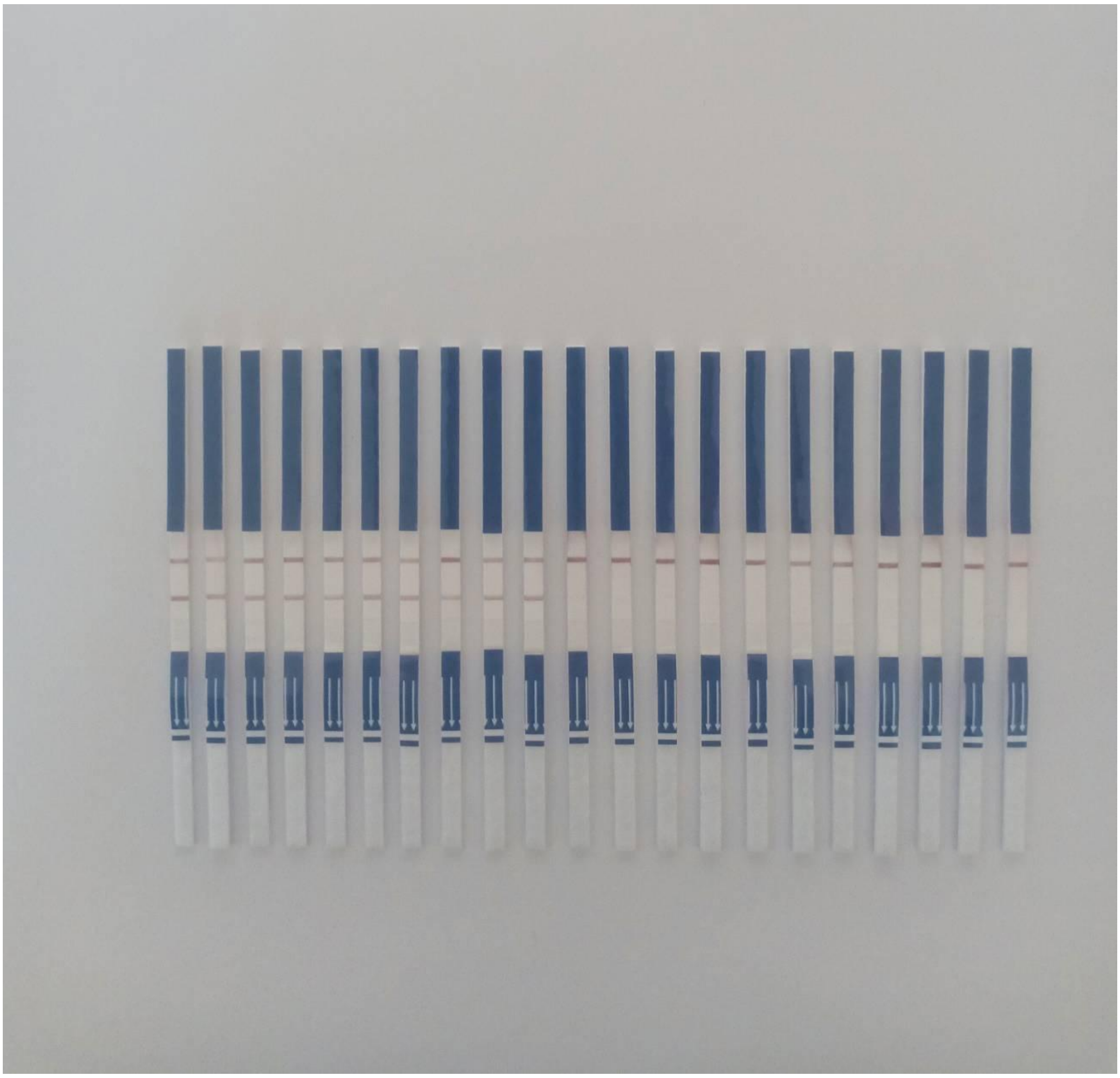

(D) The first replicate (Sample 21-40)

From left to right: Lanes 21–30: SEA-positive samples; Lanes 31–40: SEA-negative samples; Lane 41: distilled water (DW) as a negative control

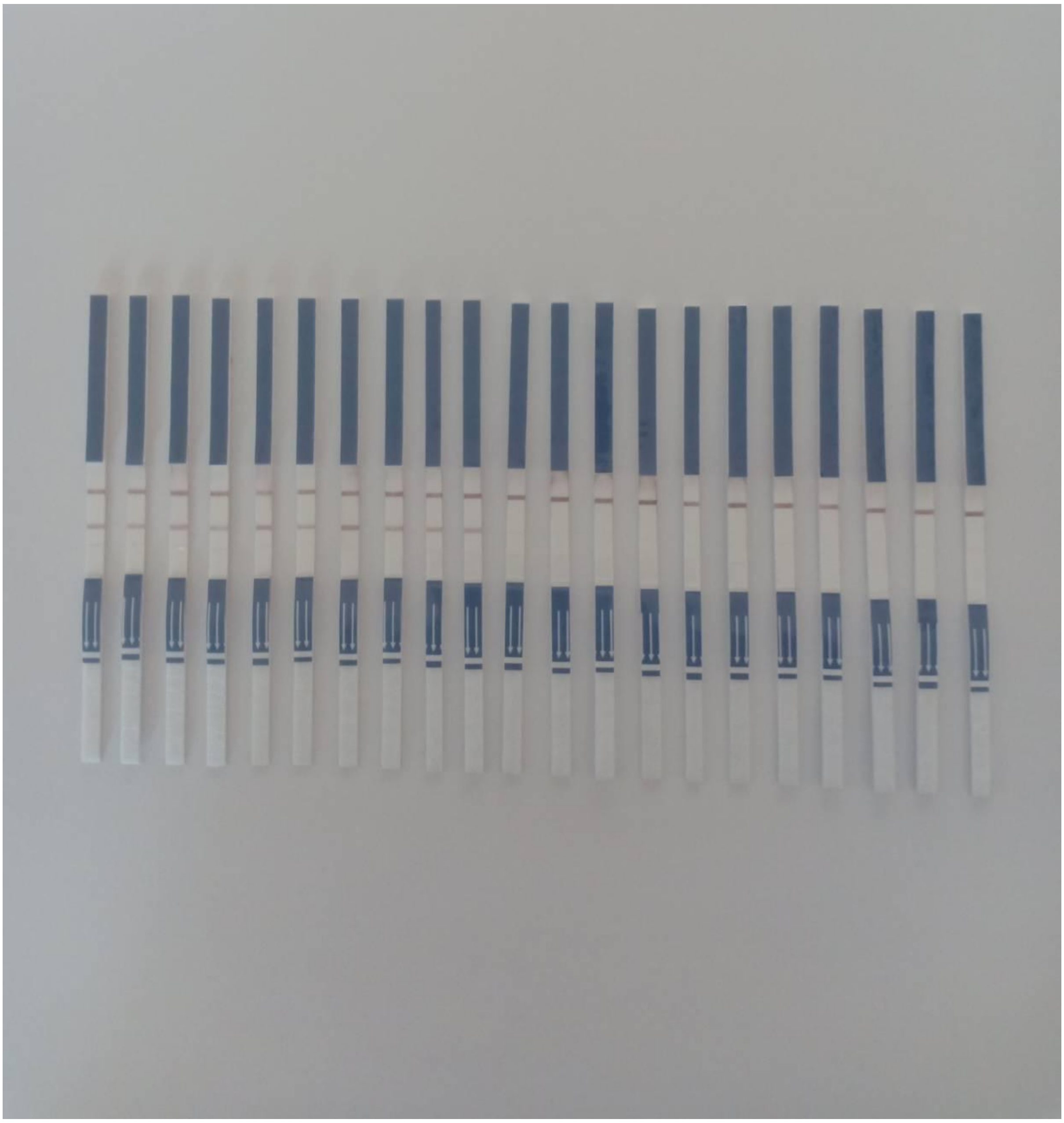

(E) The second replicate (Sample 21-40)

From left to right: Lanes 21–30: SEA-positive samples; Lanes 31–40: SEA-negative samples; Lane 41: distilled water (DW) as a negative control

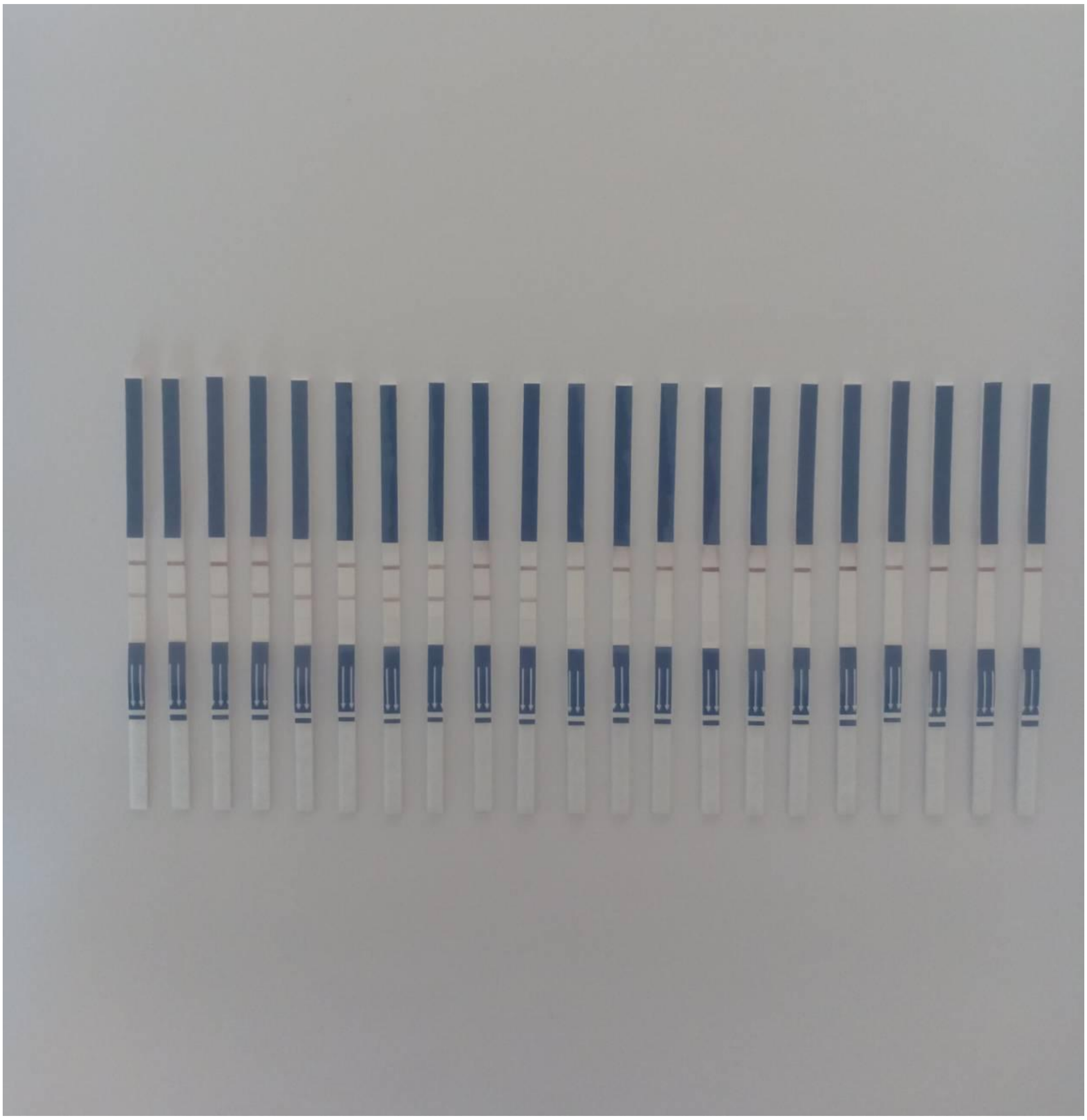

(F) The third replicate (Sample 21-40)

From left to right: Lanes 21–30: SEA-positive samples; Lanes 31–40: SEA-negative samples; Lane 41: distilled water (DW) as a negative control

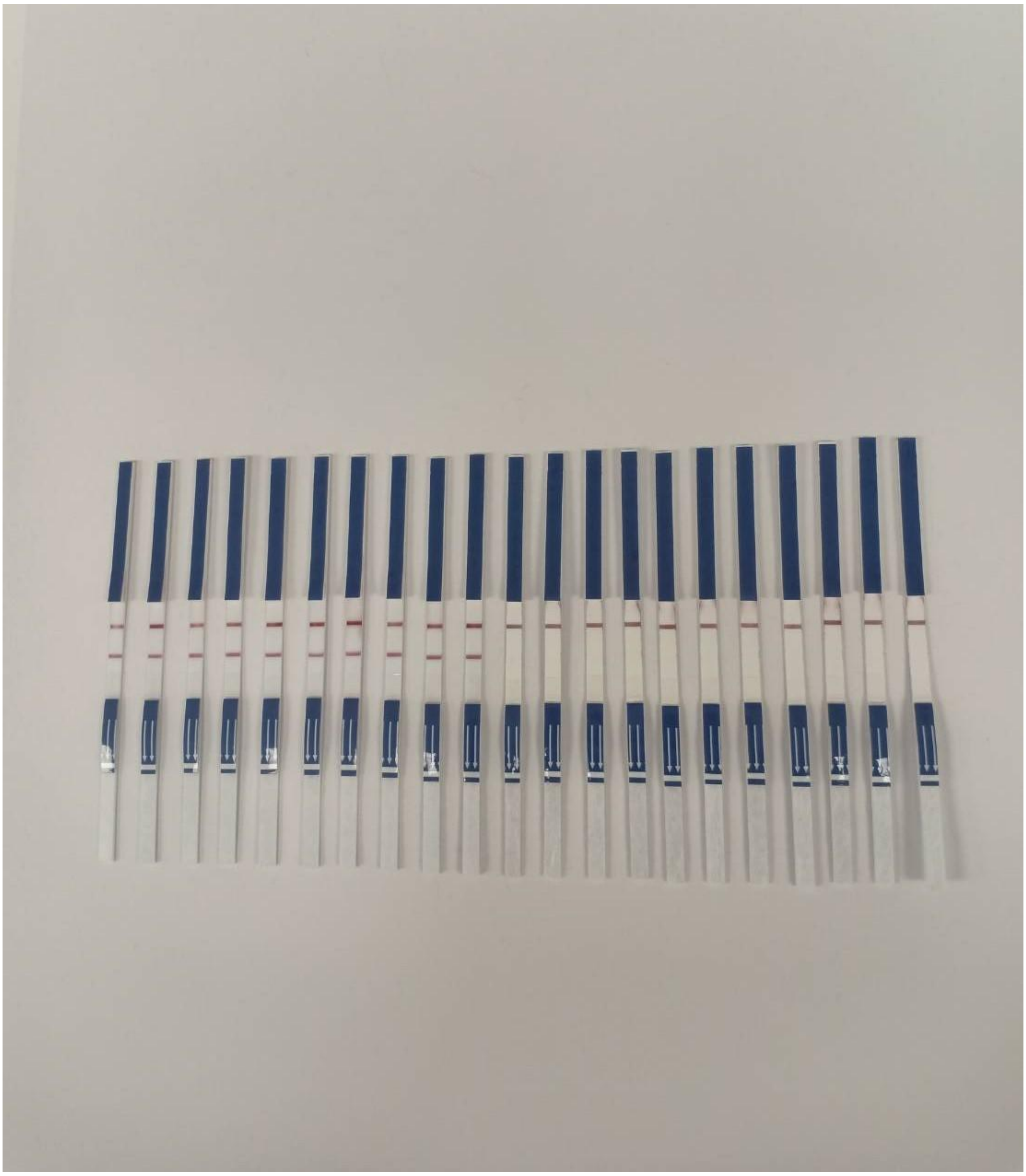

(G) The first replicate (Sample 41-50)

From left to right: Lanes 41–50: SEA-positive samples; Lanes 51–60: SEA-negative samples; Lane 61: distilled water (DW) as a negative control

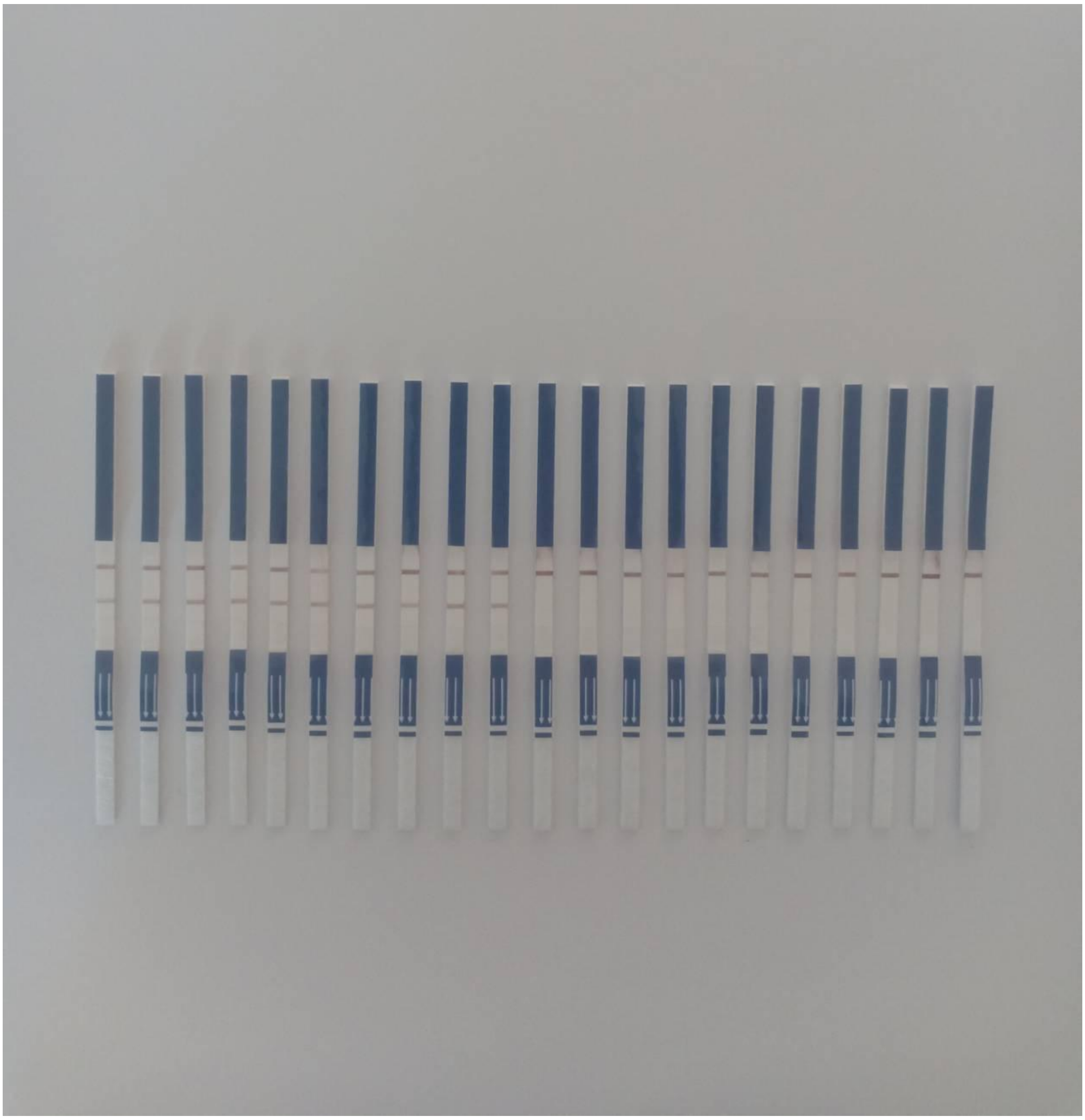

(H) The second replicate (Sample 41-50)

From left to right: Lanes 41–50: SEA-positive samples; Lanes 51–60: SEA-negative samples; Lane 61: distilled water (DW) as a negative control

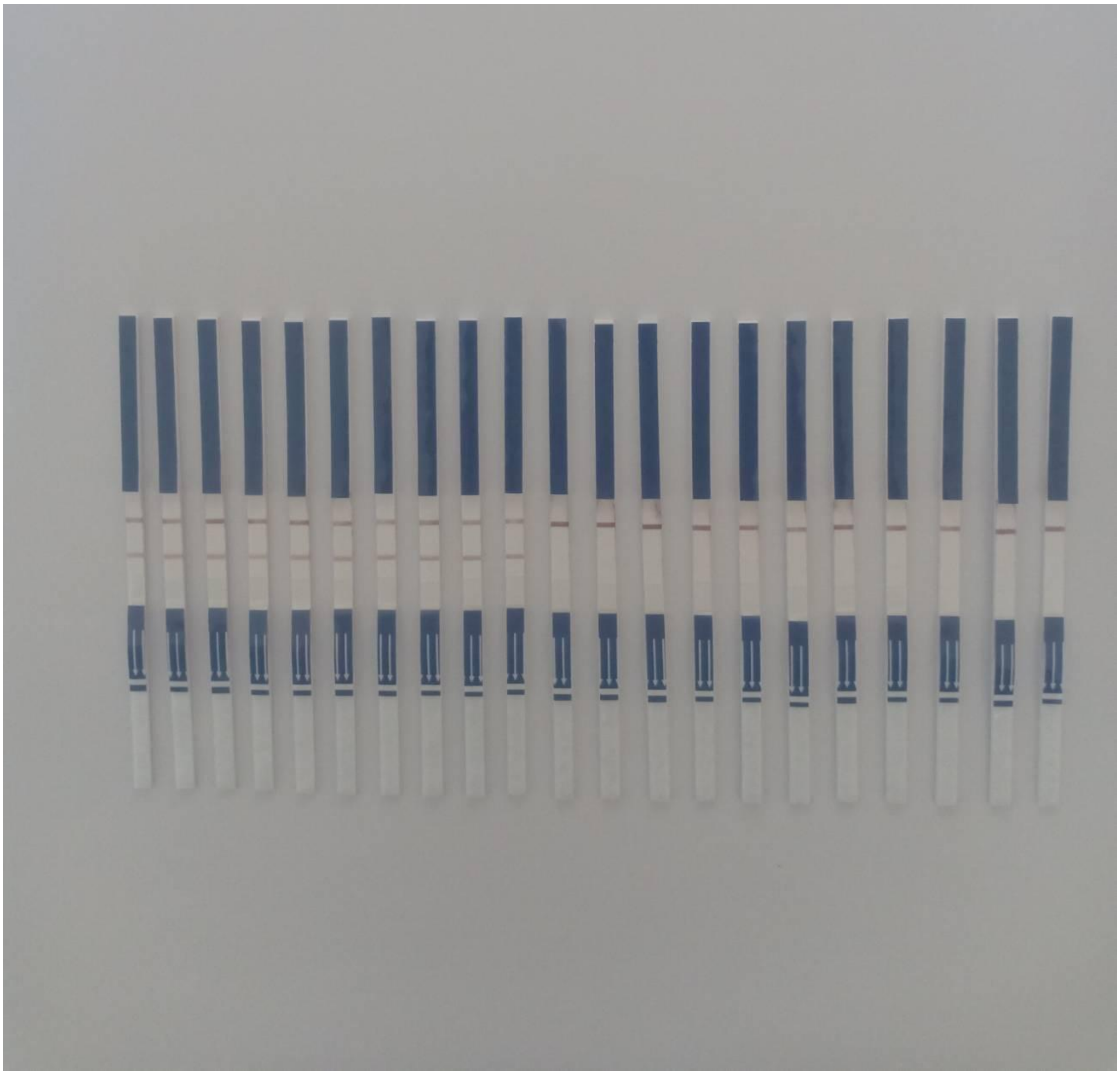

(I) The third replicate (Sample 41-50)

From left to right: Lanes 41–50: SEA-positive samples; Lanes 51–60: SEA-negative samples; Lane 61: distilled water (DW) as a negative control

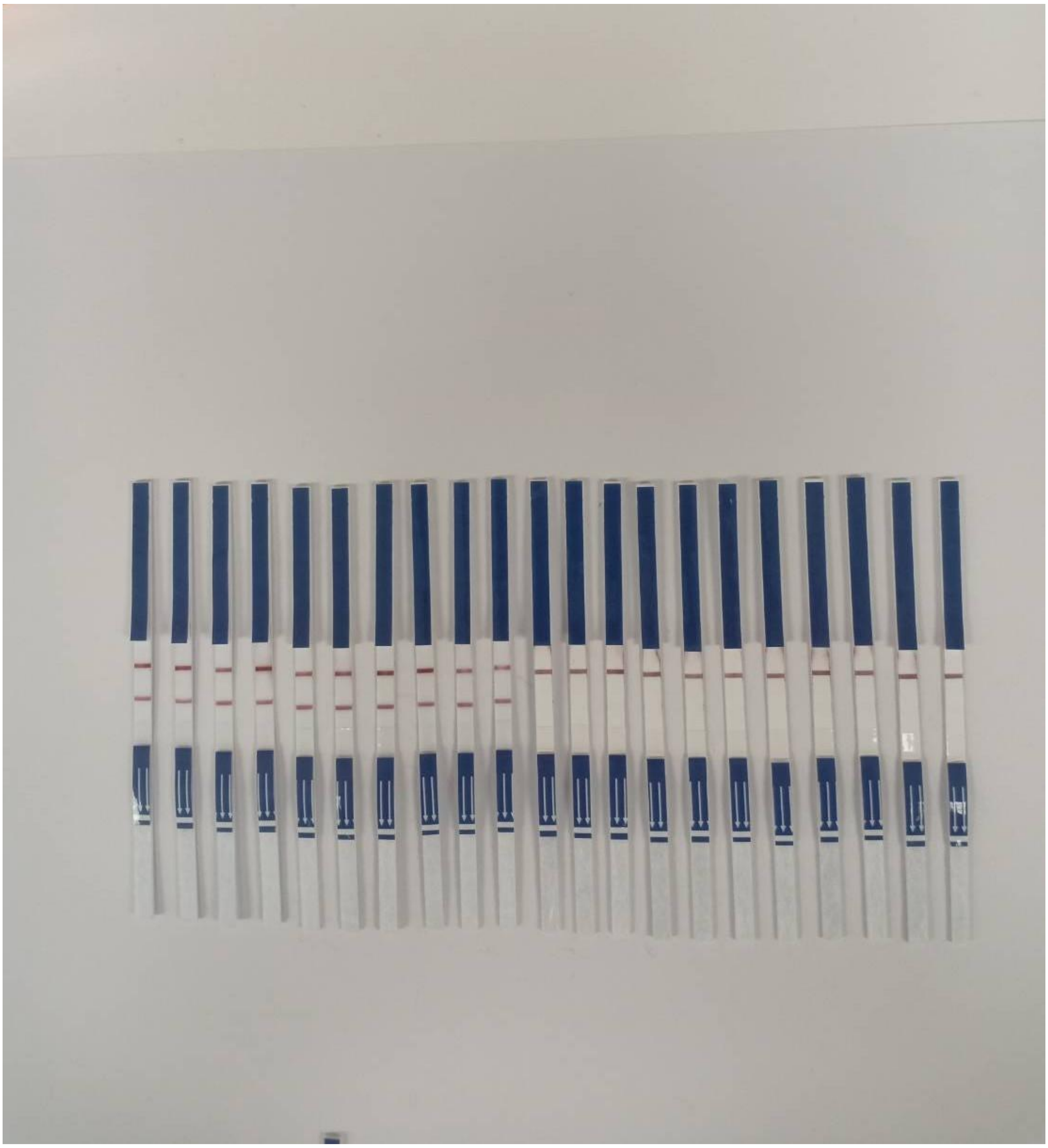

(J) The first replicate (Sample 61-80)

From left to right: Lanes 61–70: SEA-positive samples; Lanes 71–80: SEA-negative samples; Lane 81: distilled water (DW) as a negative control

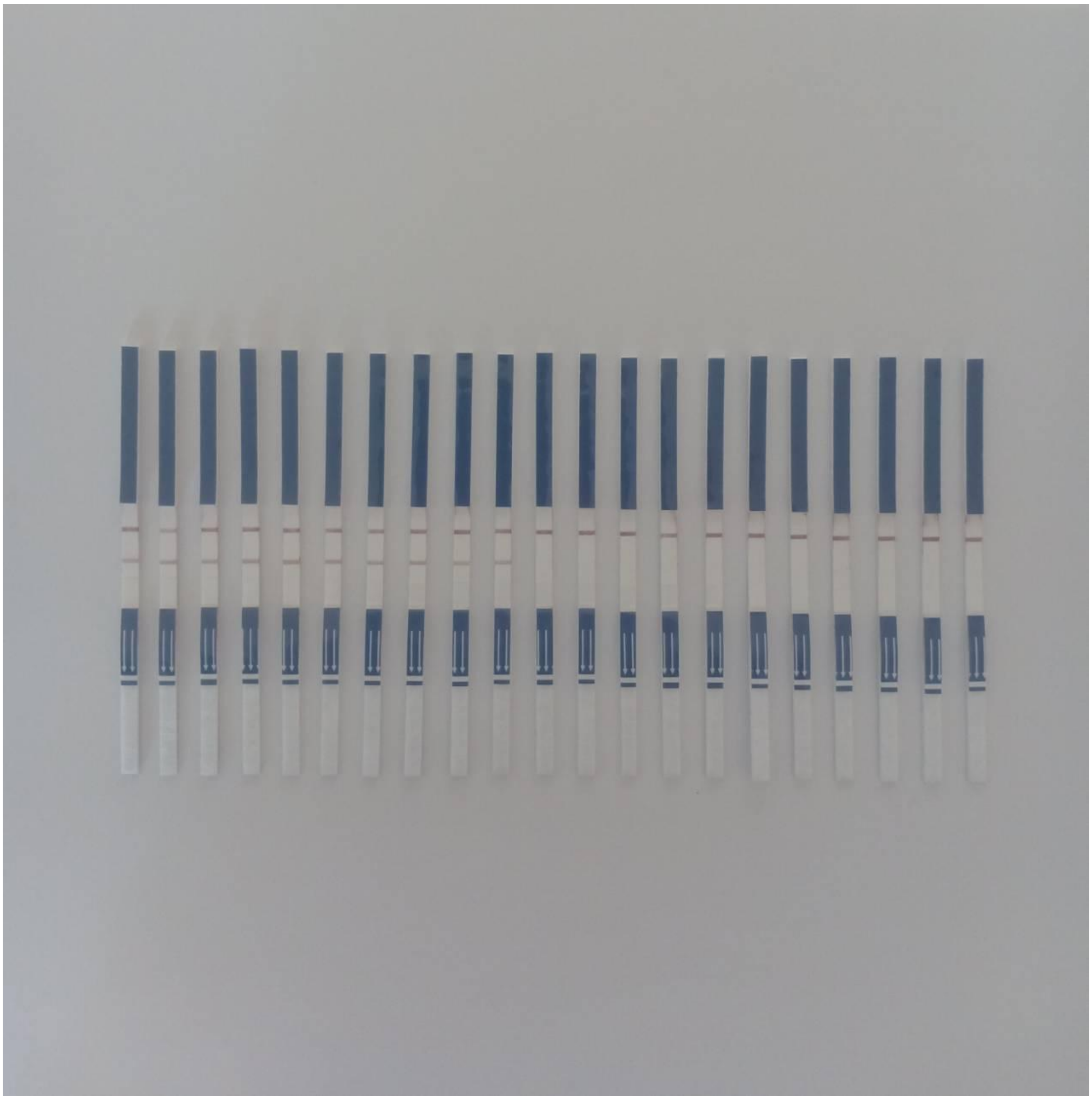

(K) The second replicate (Sample 61-80)

From left to right: Lanes 61–70: SEA-positive samples; Lanes 71–80: SEA-negative samples; Lane 81: distilled water (DW) as a negative control

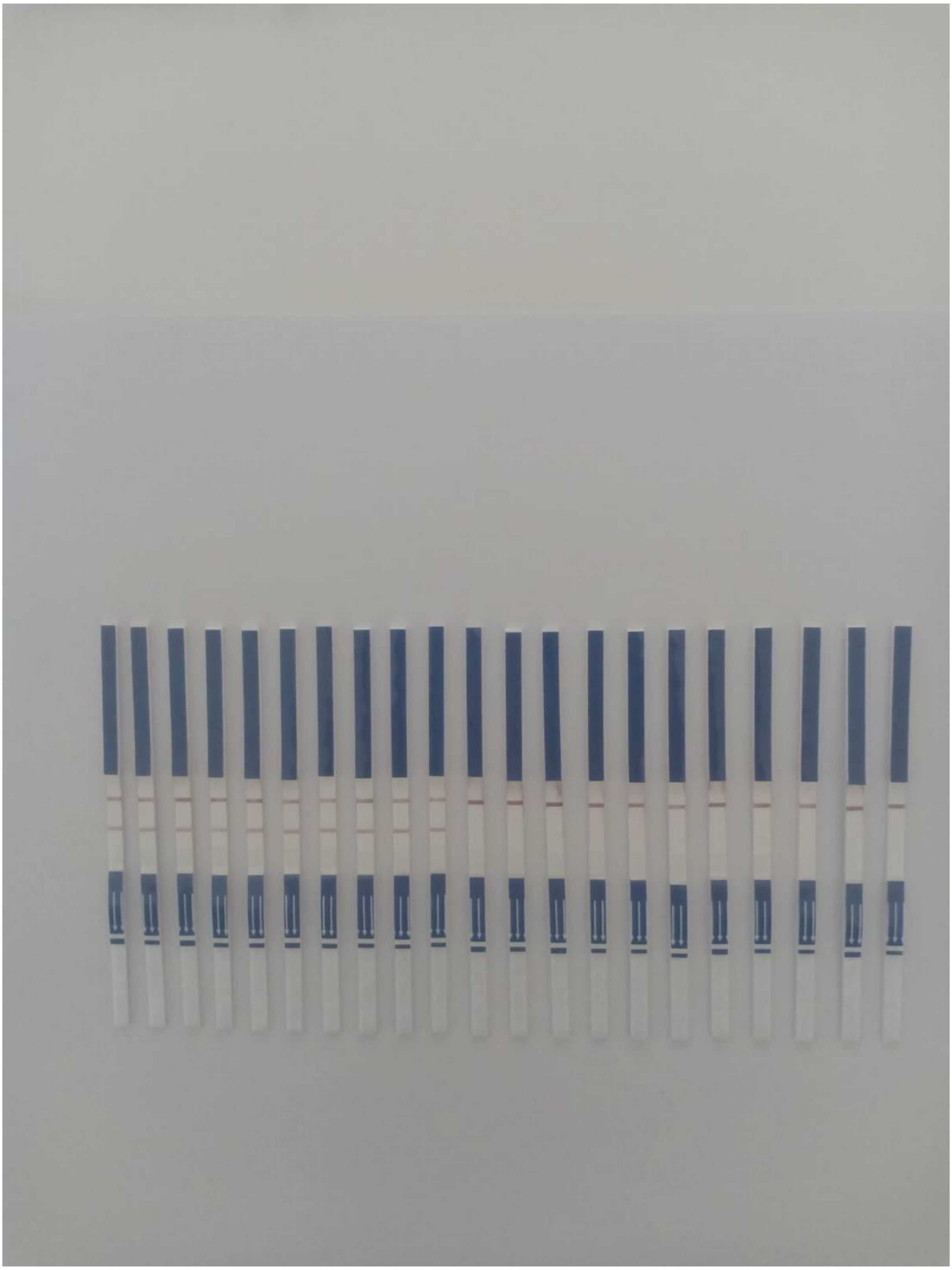

(L) The third replicate (Sample 61-80)

From left to right: Lanes 61–70: SEA-positive samples; Lanes 71–80: SEA-negative samples; Lane 81: distilled water (DW) as a negative control

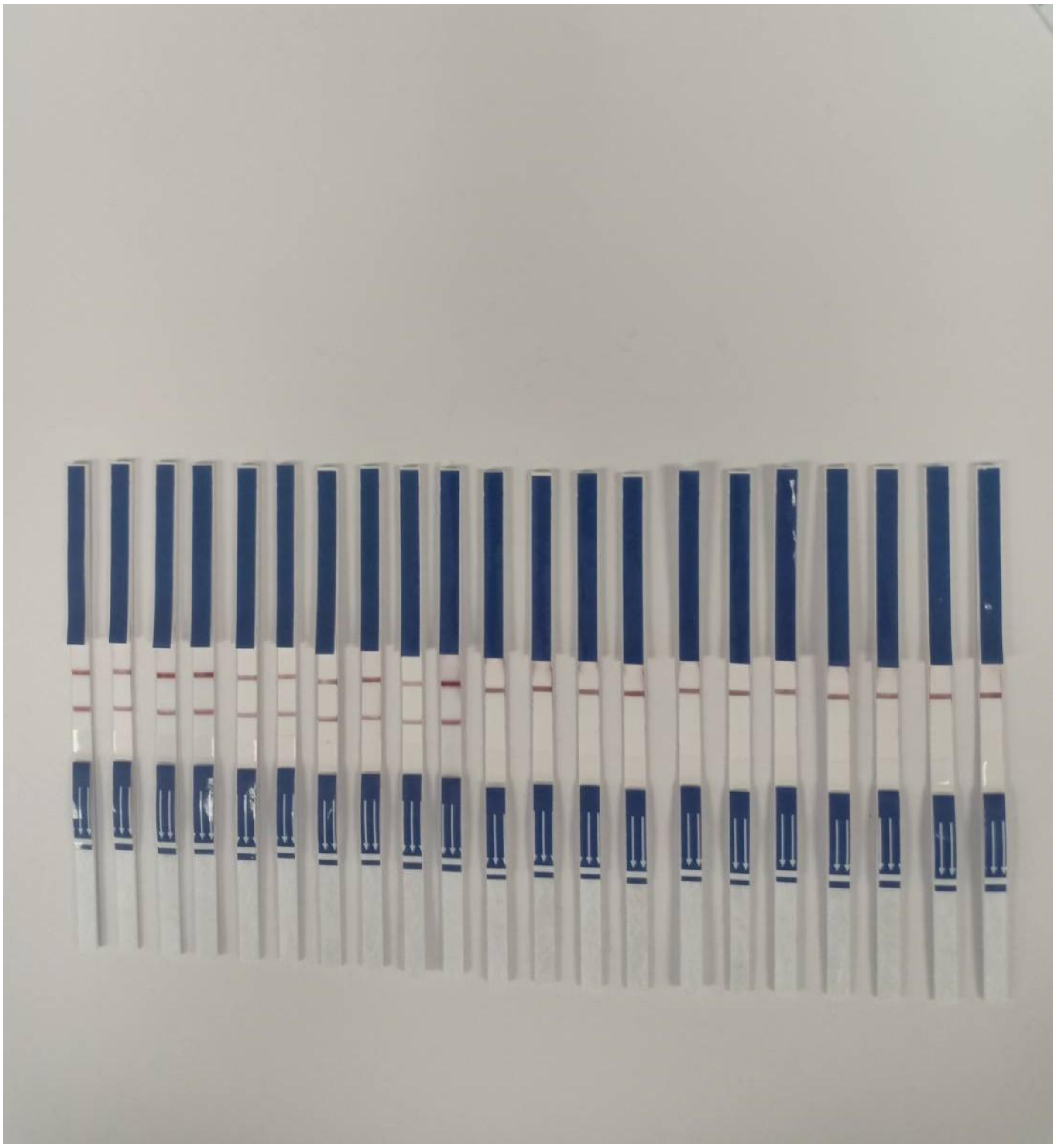

(M) The first replicate (Sample 81-96)

From left to right: Lanes 81–88: SEA-positive samples; Lanes 89–96: SEA-negative samples; Lane 97: distilled water (DW) as a negative control

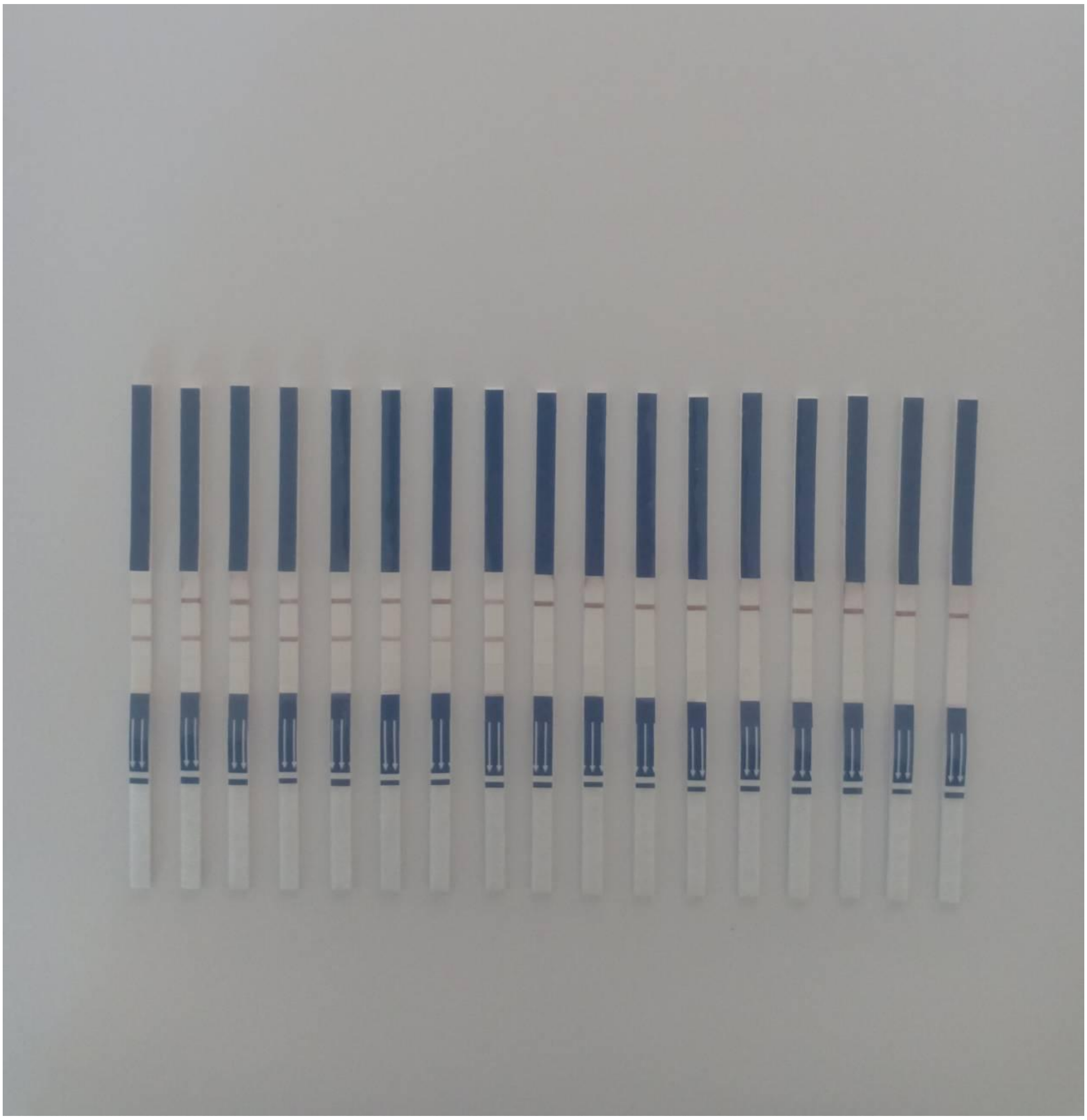

(N) The second replicate (Sample 81-96)

From left to right: Lanes 81–88: SEA-positive samples; Lanes 89–96: SEA-negative samples; Lane 97: distilled water (DW) as a negative control

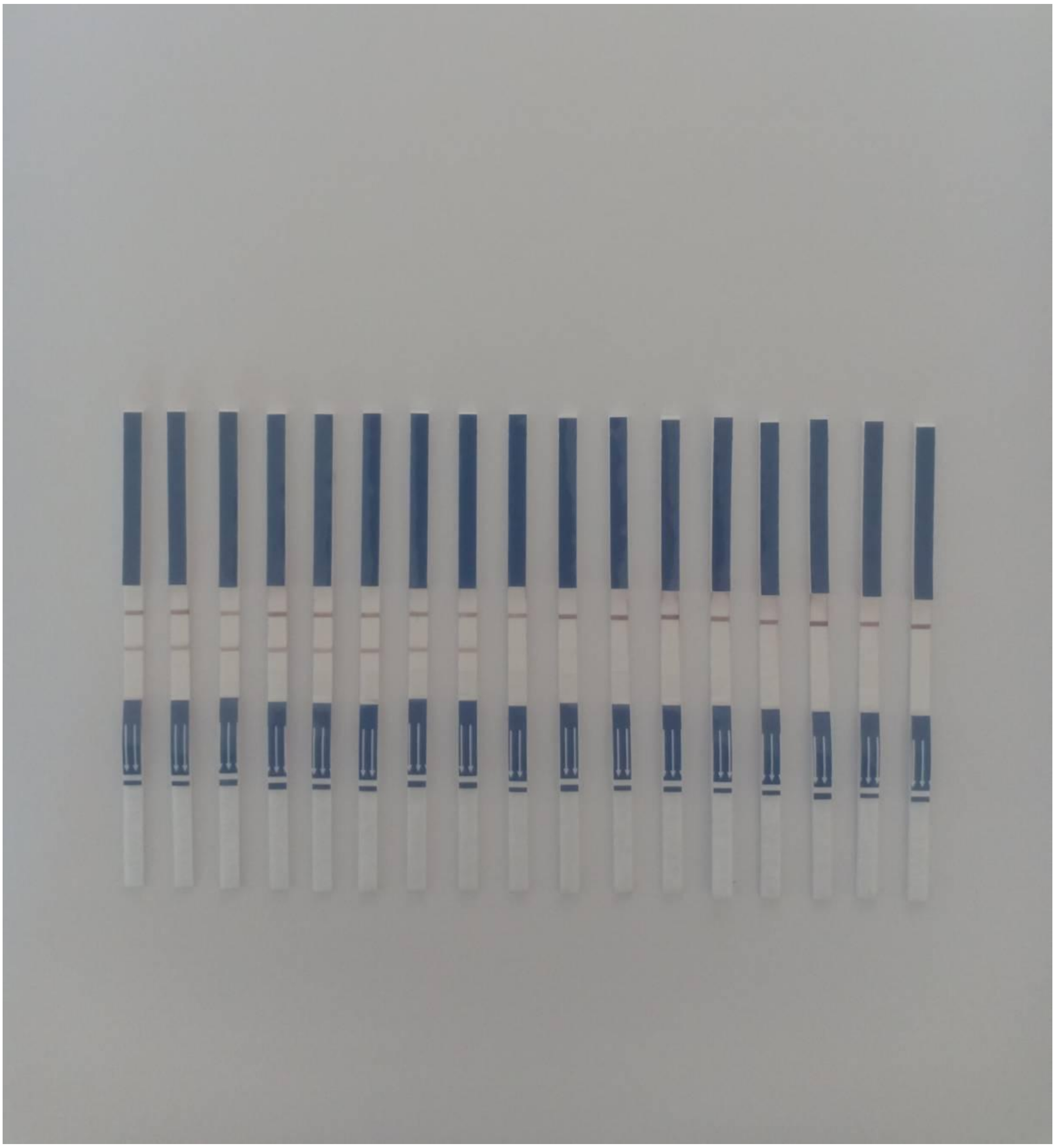

(O) The third replicate (Sample 81-96)

From left to right: Lanes 81–88: SEA-positive samples; Lanes 89–96: SEA-negative samples; Lane 97: distilled water (DW) as a negative control

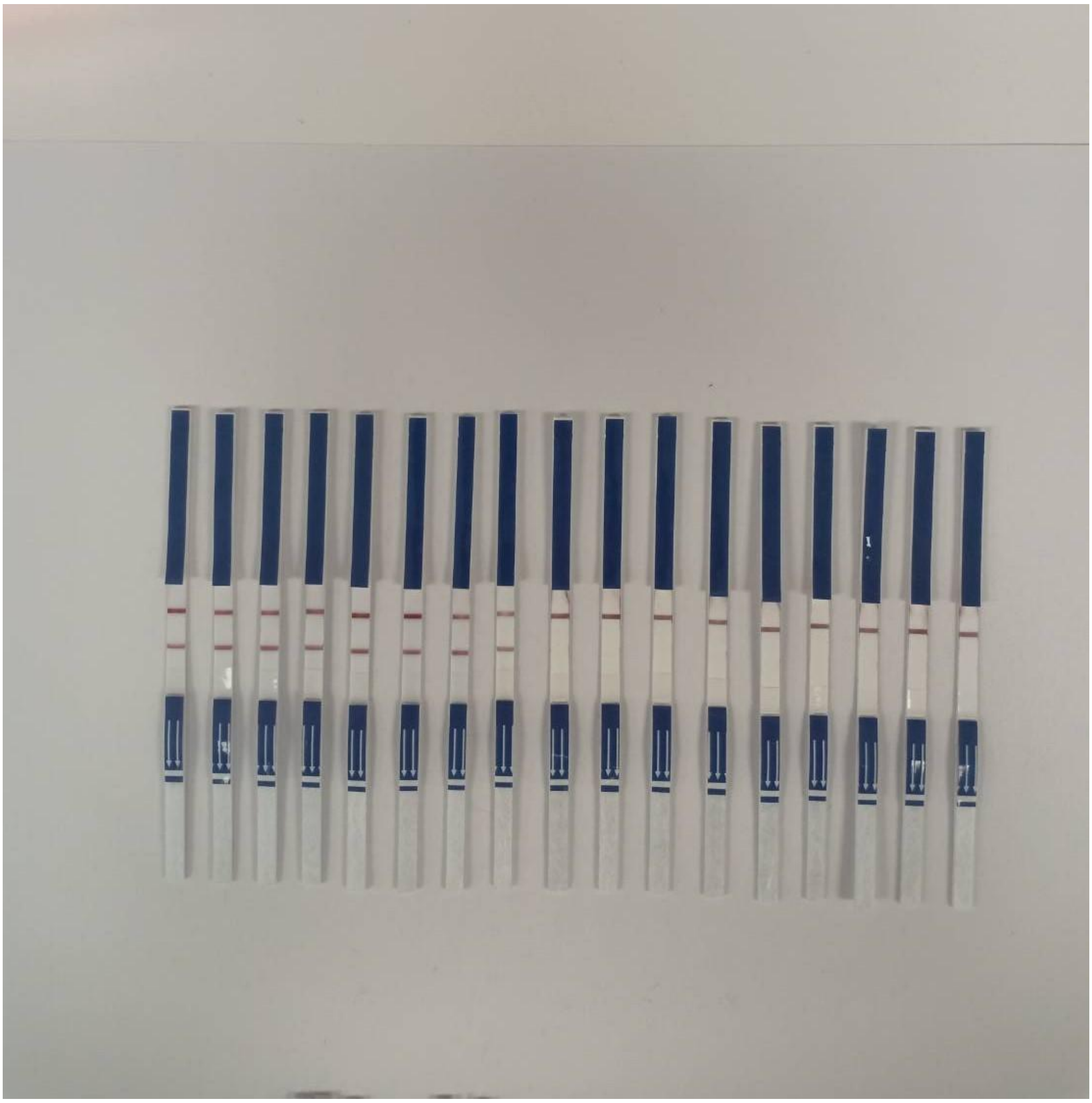

Supplement: Supplementary file 1 [file pone.0360555.s001.pdf]
